# Three Decades of Socioeconomic Inequality in India's Breastfeeding Indicators, 1993–2021

**DOI:** 10.1111/mcn.70258

**Published:** 2026-10-15

**Authors:** Rajesh Kumar Rai, Jan‐Walter De Neve, Sabri Bromage, Rockli Kim, S. V. Subramanian

**Affiliations:** ^1^ Division of Global Health Management and Policy, School of Public Health San Diego State University San Diego California USA; ^2^ Birbhum Population Project Society for Health and Demographic Surveillance Suri West Bengal India; ^3^ ImpactLoop LTD London UK; ^4^ Human Nutrition Unit, Institute of Nutrition Mahidol University Phutthamonthon Nakhon Pathom Thailand; ^5^ Department of Global Health and Population Harvard T H Chan School of Public Health Boston USA; ^6^ Heidelberg Institute of Global Health, Faculty of Medicine and University Hospital University of Heidelberg Heidelberg Germany; ^7^ Community Nutrition Unit, Institute of Nutrition Mahidol University Phutthamonthon Nakhon Pathom Thailand; ^8^ Department of Nutrition Harvard T H Chan School of Public Health Boston Massachusetts USA; ^9^ Division of Health Policy and Management, College of Health Science Korea University Seoul South Korea; ^10^ Interdisciplinary Program in Precision Public Health, Department of Public Health Sciences Graduate School of Korea University Seoul South Korea; ^11^ Harvard Center for Population and Development Studies Cambridge Massachusetts USA; ^12^ Department of Social and Behavioral Sciences Harvard T H Chan School of Public Health Boston Massachusetts USA

**Keywords:** absolute inequality, breastfeeding, exclusive breastfeeding, Global Nutrition Target, India, relative inequality

## Abstract

Although existing literature heavily focuses on the health outcomes of exclusive breastfeeding (EBF) in India, studies addressing socioeconomic inequalities across a broad spectrum of breastfeeding indicators remain scarce. We utilized five waves of National Family Health Survey data (1993–2021) to analyze temporal trends and socioeconomic inequalities, proxied by maternal education (no formal schooling, 1st–5th grade, 6th–8th grade, 9th–12th grade, and above 12th grade) and household wealth (poorest, poorer, middle, richer, and richest) across six World Health Organization‐recommended indicators: ever breastfed, early initiation of breastfeeding (EIBF), exclusive breastfeeding for the first 2 days after birth, EBF, mixed milk feeding (MixMF), and continued breastfeeding. We estimated absolute and relative inequalities between the lowest and highest categories of each socioeconomic marker, followed by the computation of the Annual Absolute Change to assess progress over time (1993–2021). Between 1993 and 2021, absolute inequality in EIBF remained stagnant between the two extreme maternal education levels. EBF disparities shifted significantly; the initial advantage held by mothers with no formal schooling in 1993 narrowed as the gap closed, a trend mirrored across household wealth quintiles where the poorest maintained a higher, albeit diminishing, lead in EBF prevalence. EIBF increased at an equal annualized rate across both the lowest and highest education strata, and MixMF rose more sharply among the poorest households. Addressing these shifting socioeconomic disparities is critical for achieving equitable breastfeeding outcomes across all strata, especially given the recent (2024) reported decline in EBF prevalence nationally.

## Key Points

- Socioeconomic disparities in breastfeeding remain deeply embedded in India, requiring targeted interventions for both disadvantaged and privileged strata.
- Stagnant “pro‐rich” gaps in early initiation of breastfeeding suggest systemic inequalities, specifically unequal access to high‐quality postpartum support and baby‐friendly healthcare standards.
- Exclusive breastfeeding (EBF) has shifted from a “pro‐poor” practice toward a state of socioeconomic convergence, indicating rapid adoption among wealthier and highly educated mothers.
- The mechanisms underlying the “pro‐poor” surge in mixed milk feeding require further investigation.
- The abrupt downturn in EBF prevalence in 2024, relative to the sustained national increase between 1993 and 2021, demands immediate intervention from policymakers.

## 1 Introduction

Often characterized as a newborn's first immunization, breastfeeding is documented to have protective effects on both infant and maternal health (Bhandari et al. 2025; Victora et al. 2016; World Health Organization WHO & the United Nations Children's Fund UNICEF 2021). Breastfeeding reduces maternal risk for metabolic disorders, cardiovascular disease, and reproductive cancers, though evidence regarding postpartum weight, depression, and osteoporosis remains inconsistent (Liu et al. 2025). For children, it protects against numerous short‐ and long‐term health risks, including respiratory and gastrointestinal infections, asthma, obesity, type 1 diabetes, childhood leukemia, and infant mortality (Liu et al. 2025; Patnode et al. 2025). The role of breastfeeding in improving maternal and child health is particularly critical in developing countries (Jiang et al. 2025; Miatton et al. 2025), where the burden of maternal and child morbidity and mortality is disproportionately high.

Although India recorded an increasing trend in exclusive breastfeeding (EBF—defined as the provision of breast milk alone, excluding all other liquids and solids, except for oral rehydration solutions and necessary vitamin, mineral, or medicinal supplements—for the first 6 months of life) between 1993 and 2021 (Rai et al. 2026), a recent estimate shows a declining prevalence of EBF—from 63.9% in 2021 (Rai et al. 2026) to 55.8% in 2024 (International Institute for Population Sciences IIPS 2026). This sharp national drop in EBF not only threatens India's ability to meet the Global Nutrition Target of ≥ 60% by 2030 (World Health Organization 2025), but it also highlights the substantial disparities that persist in breastfeeding indicators. These variations in breastfeeding practices are driven by a wide range of socioeconomic and health determinants, ranging from maternal employment and morbidity to cultural norms and lactation challenges (Aggarwal et al. 2024; Chen et al. 2024; Reddy N et al. 2023). To mitigate these barriers, particularly the socioeconomic constraints hindering breastfeeding practices, the Government of India launched the “Mothers” Absolute Affection' (MAA) program in 2016, aiming to institutionalize the promotion, protection, and support of breastfeeding within the national healthcare infrastructure (Ministry of Health and Family Welfare 2016).

Extant literature emphasizes that elevating maternal education and alleviating poverty constitute the pivotal pathways through which policymakers can effectively bolster breastfeeding practices in India (Chen et al. 2024; Sharma et al. 2024; Valappil et al. 2023). Research indicates that increased levels of maternal education significantly correlate with improved breastfeeding practices, as educated mothers are more likely to possess the health literacy and confidence required to navigate lactation challenges (Kalhor et al. 2025; Victora et al. 2016). Furthermore, the reduction of household poverty mitigates the economic pressure on mothers to return to informal or inflexible labor markets prematurely, thereby facilitating the maintenance of exclusive breastfeeding (Kalhor et al. 2025; Rollins et al. 2016). Analyzed together, higher education and financial stability create an enabling environment that bridges the gap between breastfeeding intention and long‐term practice (United Nations Children's Fund 2023). This trend toward optimal breastfeeding practices among higher socioeconomic strata aligns closely with the “diffusion of innovations” framework. In this context, socioeconomically advantaged women act as “early adopters” of public health policies or programs, whereas marginalized populations encounter systemic and structural barriers to adoption (Dearing and Cox 2018). This intersection of public health diffusion and socioeconomic disparities is clearly illustrated by the Inverse Equity Hypothesis (Victora et al. 2000), which posits that when a new public health intervention or guideline is introduced, it is initially adopted by the wealthiest and most educated segments of society, those who often already possess the lowest baseline risk of morbidity.

While associations between socioeconomic factors and breastfeeding are well‐documented, empirical data regarding progress in breastfeeding indicators across different educational and wealth brackets remain limited. Furthermore, the prevailing focus on EBF in the literature often eclipses other key indicators that are instrumental for the robust assessment of infant feeding practices in resource‐limited settings. To bridge these evidence gaps, this study utilized nationally representative data from 1993 to 2021 to evaluate temporal trends and socioeconomic (represented by maternal education and household wealth) inequalities across six WHO‐recommended breastfeeding indicators: ever breastfed (EvBF), early initiation of breastfeeding (EIBF), exclusive breastfeeding for the first 2 days after birth (EBF2D), EBF, mixed milk feeding (MixMF), and continued breastfeeding (CBF) (World Health Organization WHO and The United Nations Children's Fund UNICEF 2021). This evidence serves to inform strategic initiatives by the Ministry of Health and Family Welfare, Government of India, aimed at optimizing breastfeeding practices and narrowing socioeconomic gaps, ultimately fostering improved lifelong health trajectories for the maternal and neonatal population.

## 2 Methods

### 2.1 Dataset

This study utilizes five rounds of the nationally representative, cross‐sectional National Family Health Survey (NFHS) data, spanning 1992–2021: NFHS‐1 (1992–1993) (International Institute for Population Sciences IIPS 1995), NFHS‐2 (1998–1999) (International Institute for Population Sciences IIPS and ORC Macro 2000), NFHS‐3 (2005–2006) (International Institute for Population Sciences IIPS and Macro International 2007), NFHS‐4 (2015–2016) (International Institute for Population Sciences IIPS and ICF 2017), and NFHS‐5 (2019–2021) (International Institute for Population Sciences IIPS and ICF 2021). Although state and union territory fact sheets for select indicators are available for the sixth round of NFHS (NFHS‐6, 2023–2024) (International Institute for Population Sciences IIPS 2026), the raw microdata had not been released at the time of this study, thereby limiting our analysis to NFHS‐1 through NFHS‐5. Furthermore, beyond the aggregate estimate for EBF, the NFHS‐6 fact sheets do not provide estimates for the remaining breastfeeding indicators relevant to this study. For the purposes of this paper, these survey waves are referenced by their respective concluding years: 1993 (NFHS‐1), 1999 (NFHS‐2), 2006 (NFHS‐3), 2016 (NFHS‐4), 2021 (NFHS‐5), and 2024 (NFHS‐6). Standardized sampling frameworks ensure health and demographic estimates remain comparable across NFHS waves (Croft et al. 2023), providing a robust basis for research and policymaking. NFHSs maintained high data quality standards, consistently achieving a household response rate exceeding 95% across all rounds, while individual response rates for women remained robust, ranging between 94.5% and 96.9%. Detailed accounts of the sampling framework, instrument development, data collection protocols, weighting methodologies, and procedures for standard error estimation and quality assurance are extensively documented in the respective NFHS national reports (International Institute for Population Sciences IIPS 1995; International Institute for Population Sciences IIPS and ICF 2017; International Institute for Population Sciences IIPS and ICF 2021; International Institute for Population Sciences IIPS and Macro International 2007; International Institute for Population Sciences IIPS and ORC Macro 2000).

Across the five survey waves, the NFHSs yielded the following unweighted sample sizes for the pediatric population: NFHS‐1 (48,959), NFHS‐2 (33,026), NFHS‐3 (51,555), NFHS‐4 (259,627), and NFHS‐5 (232,920). Maternal respondents reported on breastfeeding practices for children born within specific reference periods, including the last three births since January 1988 for NFHS‐1, the last two births since January 1995 for NFHS‐2, and all births since January 2001, 2011, and 2015 for NFHS‐3, NFHS‐4, and NFHS‐5, respectively. Although all survey iterations contained the requisite data to calculate five of the six WHO‐recommended indicators, the information required to compute the EBF2D metric was available only in the NFHS‐5 dataset. A schematic representation detailing the sample derivation process is provided in the flow chart within Figure S1 of the online Supporting Information.

### 2.2 Breastfeeding Indicators

This study analyzed the coverage of six distinct binary breastfeeding indicators: EvBF, EIBF, EBF2D, EBF, MixMF, and CBF. Utilizing the child as the primary unit of analysis, the operationalization of these indicators (specifically, their respective numerators and denominators) was conducted in accordance with WHO standardized definitions (World Health Organization WHO and The United Nations Children's Fund UNICEF 2021). EvBF, EIBF, and EBF2D were assessed among children born within the preceding 24 months, measuring those ever breastfed, initiated on the breast within 1 h of birth, and exclusively breastfed for the first 48 h, respectively. EBF and MixMF were evaluated among infants aged 0–5 months based on exclusive consumption of breast milk and the inclusion of formula/animal milk, respectively, during the previous 24‐h recall period. CBF was defined as the proportion of children aged 12–23 months who received breast milk during the previous day. Although data on EBF2D (exclusive breastfeeding for the first 2 days) were only available in NFHS‐5, including this analysis was critical to determine if the practice of providing newborns with foods or liquids other than breast milk in the first few days of life, a common practice globally, is prevalent (World Health Organization WHO and The United Nations Children's Fund UNICEF 2021). Comprehensive definitions, including the derivation of specific numerators and denominators from the 1993–2021 NFHS datasets, are detailed in the published research report (Rai et al. 2026).

### 2.3 Socioeconomic Indicators: Maternal Education and Household Wealth

Maternal education and household wealth indices were developed using robust frameworks tailored and validated for the Indian socio‐demographic landscape, ensuring consistency with widely researched, locally sensitive benchmarks. The NFHS collects data on women's education in single years. Using that information, maternal educational attainment was stratified into five categories: no formal schooling (< 1 year), 1st–5th grade (1–5 years), 6th–8th grade (6–8 years), 9th–12th grade (9–12 years), and above 12th grade (> 12 years, corresponding to undergraduate or graduate coursework). This categorization reflects prevailing educational categories in India (Agrawal et al. 2019; Jain et al. 2025). Figure S2 in the online supplement presents kernel density estimates of educational attainment by number of individual years among mothers of children across five rounds of the NFHSs (1993–2021).

Household economic status was measured using a wealth index stratified into quintiles: poorest, poorer, middle, richer, and richest (International Institute for Population Sciences IIPS 1995; International Institute for Population Sciences IIPS and ICF 2017; International Institute for Population Sciences IIPS and ICF 2021; International Institute for Population Sciences IIPS and Macro International 2007; International Institute for Population Sciences IIPS and ORC Macro 2000). This widely utilized (Agrawal et al. 2019; Jain et al. 2025; Rutstein and Johnson 2004; Rutstein and Staveteig 2014) composite metric reflects a household's cumulative living standards, derived from ownership of durable assets (e.g., televisions and bicycles), housing construction materials, and access to essential infrastructure such as improved water and sanitation facilities. By serving as a proxy for long‐term economic position, the index is able to facilitate the identification of socioeconomic disparities that may be less apparent through traditional income or expenditure data (Rutstein and Johnson 2004). This approach is particularly advantageous in low‐ and middle‐income contexts such as India, where reliable nation‐wide or longitudinal financial records are often unavailable (Rutstein and Johnson 2004). Further details regarding the construction of the household wealth index are available on the official website of the Demographic and Health Surveys (DHS) Program: https://dhsprogram.com/.

### 2.4 Analytical Approach

We investigated socioeconomic inequalities in breastfeeding using a three‐pronged approach that analyzes key indicators across levels of maternal education and household wealth. We calculated absolute inequalities (prevalence difference) and relative (prevalence ratio) inequalities for all six breastfeeding indicators, stratified by socioeconomic group, over the period from 1993 to 2021. As multidimensional constructs, both measures of inequality capture variations in health outcomes between socioeconomic groups, requiring a combination of technical precision and normative judgment in metric selection (Harper et al. 2010). For example, absolute inequality (AI) in EBF for a given NFHS wave can be expressed as:

$${\mathrm{EBF}}_{\mathrm{No}\;\mathrm{schooling}\;\mathrm{or}\;\mathrm{poorest}\;\mathrm{wealth}\;\mathrm{quintile}}-{\mathrm{EBF}}_{\mathrm{Above}\;12\mathrm{th}\;\mathrm{grade}\;\mathrm{of}\;\mathrm{education}\;\mathrm{or}\;\mathrm{richest}\;\mathrm{wealth}\;\mathrm{quintile}}$$

while relative inequality (RI) could be formulated as the ratio of those prevalences –

$${\mathrm{EBF}}_{\mathrm{No}\;\mathrm{schooling}\;\mathrm{or}\;\mathrm{poorest}\;\mathrm{wealth}\;\mathrm{quintile}\;}/\;{\mathrm{EBF}}_{\mathrm{Above}\;12\mathrm{th}\;\mathrm{grade}\;\mathrm{of}\;\mathrm{education}\;\mathrm{or}\;\mathrm{richest}\;\mathrm{wealth}\;\mathrm{quintile}}$$

Furthermore, absolute inequalities (percentage point differences) were tested against a null hypothesis of zero, while relative inequalities (ratios) were explicitly tested against a null hypothesis of one, with exact *p*‐values reported for all estimates. Monitoring breastfeeding trends in India through both absolute and relative measures is essential because they provide distinct, yet equally critical, perspectives on progress toward health equity (Harper et al. 2010). An absolute measure quantifies the actual magnitude of the gap between two groups, highlighting the total “burden of inequality” and the specific effort required to close it (Braveman et al. 2011). In contrast, relative measures indicate the degree of unfairness by showing how many times more likely a favorable outcome is for one group compared to another (Harper et al. 2010). Thus, both measures compare only the extreme categories of each socioeconomic marker—mothers with no formal schooling versus those educated beyond grade 12, and children in the poorest versus the richest wealth quintile. They are therefore pairwise contrasts between extremes rather than summaries of inequality across the full socioeconomic distribution: they draw on no information from the intermediate education categories or wealth quintiles and are unaffected by how the population is distributed across them.

As a next step, we computed the Annual Absolute Change (AAC) (Subramanian et al. 2023) to assess progress across breastfeeding indicators by socioeconomic groups over time (between 1993 and 2021), formulated as follows:

$$\mathrm{AAC}=[(P_{t}-P_{t-n})/n]$$

where $P_{t}$ is the coverage of breastfeeding at time *t* and $P_{t-n}$ denotes the coverage of breastfeeding n years before time *t*, with *n* representing the inter‐survey gap in years between two consecutive NFHSs. Positive AAC values signify upward trends in breastfeeding prevalence, which constitute improvements for most indicators; however, for MixMF, such an increase represents a worsening of the practice. Negative values reflect a downward trend or decline. AAC was evaluated across five distinct intervals of varying duration: 1993–1999 (6 years), 1999–2006 (7 years), 2006–2016 (10 years), and 2016–2021 (5 years), as well as a cumulative 30‐year period spanning 1993– 2021 (Subramanian et al. 2023). AAC was interpreted in terms of annualized percentage point changes.

To further confirm the influence of maternal education and household economic status on breastfeeding indicators, we performed a multivariable analysis using a modified Poisson regression model with robust error variance (Zou 2004). For common binary outcomes, this approach directly estimates relative risks, avoiding the overestimation of odds ratios in logistic regression and the convergence failures of log‐binomial models, while using robust standard errors. Data from all five NFHS waves were pooled for the model, and survey rounds were included as controls alongside other potential covariates to isolate the net association between socioeconomic factors and breastfeeding indicators. Guided by existing literature (Aggarwal et al. 2024; Chen et al. 2024; Reddy N et al. 2023; Valappil et al. 2023), potential control variables, in addition to maternal education and household wealth, included maternal age, mass media exposure (if the mother watched television or listened to the radio every week), number of antenatal care (ANC) visits (< 4 vs. ≥ 4), place of delivery (home vs. health facility), birth order of the child (first, second, or third/higher), child's age (in months), sex of the child, household size, social group (Scheduled Castes, Scheduled Tribes, Others), religion (Hinduism, Islam, Others), locality of residence (urban vs. rural), and region of residence (North, South, East, West, Central, and Northeast). Social groups were categorized in alignment with the constitutional classifications defined by the Government of India. Variables were selected based on their availability across all five survey rounds. All analyses were performed in Stata (version 18.0) using the *svy* prefix command to adjust for the complex sample survey design (StataCorp 2023) and ensure that estimates were representative of the target populations.

We utilized complete case analysis for all statistical procedures. In the descriptive and inequality analyses, a child contributed to an indicator's denominator only if there were no missing data for the variables comprising the indicator, nor for the relevant stratifying variables (maternal education or household wealth). Exclusions were indicator‐specific, so analytic sample sizes vary across indicators and waves. Multivariable models excluded observations missing the outcome or any covariate. No imputation or inverse‐probability weighting for missingness was used. Missingness was modest overall and concentrated in the ever‐breastfed and early‐initiation indicators in 2016 and 2021. Because complete‐case analysis is unbiased only if missingness is unrelated to the outcome conditional on covariates, and non‐response may itself vary with maternal education or household wealth, we treat this as a limitation, discussed below.

### 2.5 Ethics Statement

Prior to conducting the National Family Health Survey (NFHS), all procedures involving research participants were approved by the Institutional Ethics Review Board constituted by the Ministry of Health and Family Welfare, Government of India. The DHS Program (https://dhsprogram.com/) hosts the NFHS datasets with all survey participant identifiers removed, allowing the use of the data to be exempt from a full institutional ethics review.

## 3 Results

### 3.1 Descriptive Statistics

Maternal education levels rose significantly during the study period; the mean years of schooling increased from 2.96 years (standard deviation or SD: 4.33) in 1993 to 6.84 years (SD: 5.31) in 2021 (authors' estimates based on NFHS data for married women aged 15–49 years as the denominator). While breastfeeding rates for EvBF and CBF remained robust between 1993 and 2021, with EvBF exceeding 95% and CBF staying above 75%, the period was marked by a dramatic rise in early initiation (9.6%–41.2%) and EBF (43.5%–63.9%), though these gains were tempered by a 9‐percentage‐point increase in MixMF (Figure S3, online supplement). Notably, the recently released NFHS‐6 (2024) fact sheets reveal a concerning decline in the prevalence of EBF, which dropped to 55.8% (International Institute for Population Sciences IIPS 2026) from 63.9% in NFHS‐5 (Rai et al. 2026).

### 3.2 Inequalities in Breastfeeding Indicators Between Socioeconomic Extremes

Trends in the prevalence of breastfeeding indicators (1993–2021) were stratified by maternal education and household wealth (Tables S1 and S2, online supplement). These trends, visualized in Figures 1 and 2, summarize both absolute and relative inequalities (Table 1).

**Figure 1:**
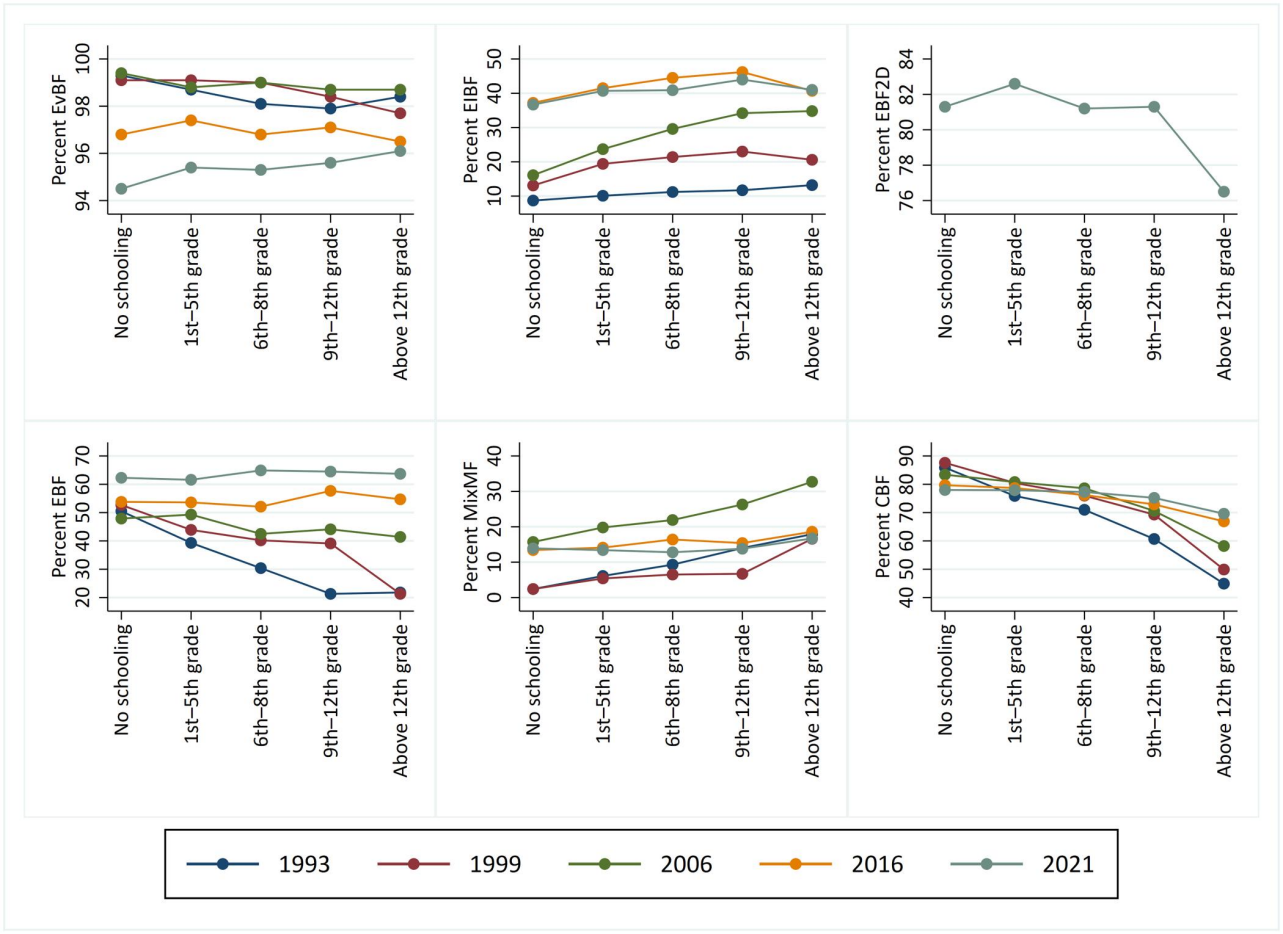
Temporal trends in breastfeeding indicators by maternal education, 1993–2021. EvBF, ever breastfed; EIBF, early initiation of breastfeeding; EBF2D, exclusively breastfed for the first 2 days after birth; EBF, exclusive breastfeeding under 6 months; MixMF, mixed feeding under 6 months; CBF, continued breastfeeding 12–23 months.

**Figure 2:**
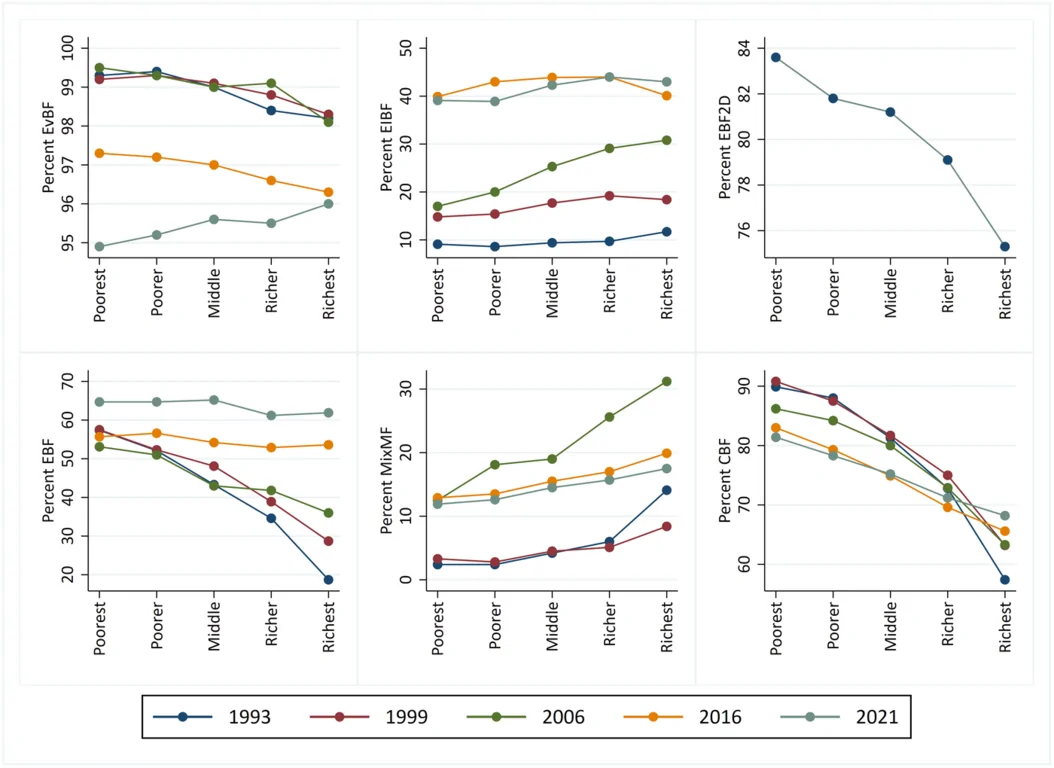
Temporal trends in breastfeeding indicators by household wealth, 1993–2021. EvBF, ever breastfed; EIBF, early initiation of breastfeeding; EBF2D, exclusively breastfed for the first 2 days after birth; EBF, exclusive breastfeeding under 6 months; MixMF, mixed feeding under 6 months; CBF, continued breastfeeding 12–23 months.

**Table 1:** Absolute and relative inequalities between the lowest socioeconomic strata (no formal schooling or poorest quintile) and the highest strata (education beyond 12th grade or wealthiest quintile) across six breastfeeding indicators: EvBF, EIBF, EBF2D, EBF, MixMF, and CBF, categorized by NFHS survey year.

	EvBF	EIBF	EBF2D	EBF	MixMF	CBF
**Maternal education**						
Absolute inequality (95% CI) p						
1993	0.9 (−0.17, 1.99) 0.100	−4.5 (−7.50, −1.64) 0.002	rina	28.7 (20.91, 36.37) < 0.001	−15.5 (−22.14, −8.68) < 0.001	41.0 (34.88, 47.03) < 0.001
1999	1.4 (0.07, 2.79) 0.040	−7.5 (−10.65, −4.18) < 0.001	rina	31.4 (23.79, 39.00) < 0.001	−14.2 (−20.44, −7.90) < 0.001	37.7 (32.01, 43.36) < 0.001
2006	0.7 (−0.08, 1.47) 0.077	−18.7 (−22.16, −15.29) < 0.001	rina	6.5 (−1.17, 14.15) 0.097	−17.0 (−24.01, −10.03) < 0.001	25.2 (20.12, 30.25) < 0.001
2016	0.3 (−0.41, 1.00) 0.407	−3.5 (−5.11, −1.79) < 0.001	rina	−0.9 (−4.20, 2.51) 0.621	−5.2 (−7.58, −2.87) < 0.001	12.8 (10.57, 15.05) < 0.001
2021	−1.6 (−2.13, −0.94) < 0.001	−4.3 (−5.77, −2.69) < 0.001	4.8 (3.58, 6.16) < 0.001	−1.4 (−4.27, 1.47) 0.339	−2.8 (−4.91, −0.58) 0.013	8.4 (6.50, 10.40) < 0.001
β (95% CI) p^a^	−0.08 (−0.20, 0.04) 0.114	0.09 (−0.49, 0.68) 0.650	rina	−1.28 (−1.97, −0.58) 0.010	0.49 (0.19, 0.79) 0.014	−1.24 (−1.49, −0.99) 0.001
Relative inequality (95% CI) p						
1993	1.01 (0.998, 1.020) 0.103	0.66 (0.51, 0.80) < 0.001	rina	2.32 (1.51, 3.11) 0.001	0.13 (0.08, 0.20) < 0.001	1.91 (1.66, 2.17) < 0.001
1999	1.01 (1.00, 1.03) 0.042	0.64 (0.53, 0.74) < 0.001	rina	2.47 (1.62, 3.32) 0.001	0.14 (0.08, 0.21) < 0.001	1.76 (1.56, 1.95) < 0.001
2006	1.01 (0.999, 1.015) 0.079	0.46 (0.41, 0.51) < 0.001	rina	1.16 (0.95, 1.37) 0.145	0.48 (0.36, 0.59) < 0.001	1.43 (1.31, 1.55) < 0.001
2016	1.00 (0.996, 1.010) 0.408	0.91 (0.88, 0.95) < 0.001	rina	0.98 (0.92, 1.05) 0.617	0.72 (0.62, 0.82) < 0.001	1.19 (1.15, 1.23) < 0.001
2021	0.98 (0.98, 0.99) < 0.001	0.90 (0.86, 0.93) < 0.001	1.06 (1.05, 1.08) < 0.001	0.98 (0.93, 1.02) 0.333	0.83 (0.72, 0.95) 0.006	1.12 (1.09, 1.15) < 0.001
β (95% CI) p^a^	−0.0009 (−0.0021, 0.0002) 0.084	0.011 (−0.002, 0.024) 0.076	rina	−0.06 (−0.09, −0.02) 0.014	0.03 (0.02, 0.04) 0.002	−0.03 (−0.04, −0.02) 0.001
**Household wealth**						
Absolute inequality (95% CI) p						
1993	1.1 (0.48, 1.58) < 0.001	−2.6 (−4.17, −1.13) 0.001	rina	38.7 (34.29, 43.11) < 0.001	−11.7 (−14.57, −8.78) < 0.001	32.5 (29.49, 35.49) < 0.001
1999	0.9 (0.23, 1.53) 0.008	−3.6 (−5.59, −1.54) 0.001	rina	28.8 (23.69, 33.92) < 0.001	−5.1 (−7.69, −2.47) < 0.001	27.6 (24.51, 30.71) < 0.001
2006	1.4 (0.69, 1.98) < 0.001	−13.8 (−16.2, −11.4) < 0.001	rina	17.1 (11.42, 22.74) < 0.001	−18.7 (−23.49, −13.94) < 0.001	22.9 (19.48, 26.31) < 0.001
2016	1.0 (0.32, 1.69) 0.004	−0.2 (−1.78, 1.35) 0.790	rina	2.1 (−0.99, 5.22) 0.182	−7.0 (−9.40, −4.62) < 0.001	17.4 (15.36, 19.52) < 0.001
2021	−1.1 (−1.70, −0.44) 0.001	−3.9 (−5.45, −2.37) < 0.001	8.3 (6.98, 9.55) < 0.001	2.8 (−0.19, 5.78) 0.066	−5.6 (−7.95, −3.31) < 0.001	13.2 (11.30, 15.10) < 0.001
β (95% CI) p^a^	−0.06 (−0.18, 0.07) 0.247	0.04 (−0.39, 0.48) 0.779	rina	−1.36 (−1.88, −0.84) 0.004	0.15 (−0.27, 0.57) 0.331	−0.66 (−0.74, −0.59) < 0.001
Relative inequality (95% CI) p						
1993	1.01 (1.00, 1.02) < 0.001	0.78 (0.66, 0.89) < 0.001	rina	3.07 (2.54, 3.59) < 0.001	0.17 (0.09, 0.25) < 0.001	1.57 (1.49, 1.64) < 0.001
1999	1.01 (1.00, 1.02) 0.009	0.80 (0.71, 0.90) < 0.001	rina	2.00 (1.71, 2.29) < 0.001	0.39 (0.20, 0.59) < 0.001	1.44 (1.37, 1.50) < 0.001
2006	1.01 (1.01, 1.02) < 0.001	0.55 (0.49, 0.61) < 0.001	rina	1.48 (1.27, 1.68) < 0.001	0.40 (0.30, 0.50) < 0.001	1.36 (1.29, 1.43) < 0.001
2016	1.01 (1.00, 1.02) 0.004	1.00 (0.96, 1.03) 0.789	rina	1.04 (0.98, 1.10) 0.194	0.65 (0.56, 0.74) < 0.001	1.27 (1.23, 1.30) < 0.001
2021	0.99 (0.982, 0.995) < 0.001	0.91 (0.88, 0.94) < 0.001	1.11 (1.09, 1.13) < 0.001	1.05 (0.995, 1.094) 0.075	0.68 (0.58, 0.78) < 0.001	1.19 (1.16, 1.23) < 0.001
β (95% CI) p^a^	−0.001 (−0.002, 0.001) 0.249	0.007 (−0.005, 0.019) 0.158	rina	−0.07 (−0.12, −0.01) 0.028	0.018 (0.010, 0.025) 0.004	−0.013 (−0.016, −0.009) 0.002

Abbreviations: β, coefficient; CI, confidence interval; EvBF, ever breastfed; EIBF, early initiation of breastfeeding; EBF2D, exclusively breastfed for the first two days after birth; EBF, exclusive breastfeeding under six months; MixMF, mixed feeding under six months; CBF, continued breastfeeding 12‐23 months; NFHS, National Family Health Survey; p, level of significance; rina, required information not available.

^a^ Trends for breastfeeding inequality indicators were calculated using Ordinary Least Squares (OLS) regression with robust standard errors, where inequality estimates were used as the outcome variable and the survey year (1993, 1999, 2006, 2016, and 2021) was treated as the covariate.

For EIBF, absolute inequality between extreme educational strata remained virtually unchanged from 1993 (−4.5 percentage points) to 2021 (−4.3 percentage points), consistently favoring more educated mothers (see Table 1, Figure 1, and Table S1 in the online supplement). In contrast, in 1993, EBF exhibited a significant disparity in favor of mothers with no formal schooling (AI: 28.7; RI: 2.32). By 2021, this gap had effectively closed, shifting toward a marginal advantage for the highest education group (AI: −1.4; RI: 0.98). A declining trend in both AI (*β*: −1.28) and RI (*β*: −0.06) was observed for EBF between 1993 and 2021. Socioeconomic disparities in MixMF narrowed substantially over the study period, particularly among children of more educated mothers (*β* for trend – AI: 0.49, RI: 0.03). Finally, while CBF also favored children of mothers with no formal schooling, the disparity between the educational extremes (no schooling vs. a 12th‐grade education or higher) contracted significantly between 1993 (AI: 41.0; RI: 1.91) and 2021 (AI: 8.4; RI: 1.12).

The disparities in breastfeeding indicators between children from the poorest and richest households largely mirrored the trends observed across maternal education levels (no formal schooling vs. at least a 12th‐grade education) (see Table 1, Figure 2, and Table S2 in the online supplement). Regarding EBF, children from the poorest wealth quintile retained a higher prevalence than those from the richest quintile, although the disparity narrowed significantly from 1993 (AI: 38.7; RI: 3.07) to 2021 (AI: 2.8; RI: 1.05). Similarly, a persistent socioeconomic gradient was observed for MixMF and CBF, with both indicators consistently favoring children from the poorest households throughout the 1993–2021 study period.

### 3.3 Trends in Socioeconomic Inequalities Over Time (1993–2021)

The AAC, representing the annualized shifts in breastfeeding indicators from 1993 to 2021 by maternal education and household wealth, is presented in Tables S3 and S4, respectively (online supplement). From 1993 to 2021, the annualized increase (AAC) in EIBF prevalence was 0.93 percentage points for both the lowest and highest maternal education strata. In contrast, EBF showed a steeper upward trend among children of more educated mothers. Conversely, within the no‐schooling group, MixMF prevalence increased (AAC: 0.38 percentage points) while CBF decreased (AAC: −0.26 percentage points). Consistent with the trends by maternal educational attainment, a positive economic gradient by household wealth was observed in the AAC for EBF. Notably, the AAC of increase in MixMF prevalence was higher among children in the poorest wealth quintile than among their wealthiest counterparts.

### 3.4 Association With Maternal Education and Household Wealth

Findings from the regression analysis confirming the roles of maternal education and household wealth in breastfeeding practices are presented in Tables S5–S7 (online supplement), while Figure 3 presents the primary findings. Compared to children of mothers with no formal schooling, the prevalence of EBF2D and CBF was significantly lower among those whose mothers were educated beyond the 12th grade (relative risk or RR: 0.97; 95% CI: 0.96, 0.99; *p* < 0.001 and RR: 0.94; 95% CI: 0.93, 0.96; *p* < 0.001, respectively). Conversely, a positive association was observed for MixMF (RR: 1.30; 95% CI: 1.20, 1.42; *p* < 0.001). Similarly, regarding household wealth, children from the richest households exhibited a significantly lower prevalence of EvBF, EIBF, EBF2D, EBF, and CBF than those from the poorest households (*p* < 0.001), whereas the opposite trend was observed for MixMF (*p* < 0.001). Furthermore, the multivariable regression model suggests that, compared to 1993, there was a gradual increase in the prevalence of EBF, as evidenced by an increasing RR in each subsequent year.

**Figure 3:**
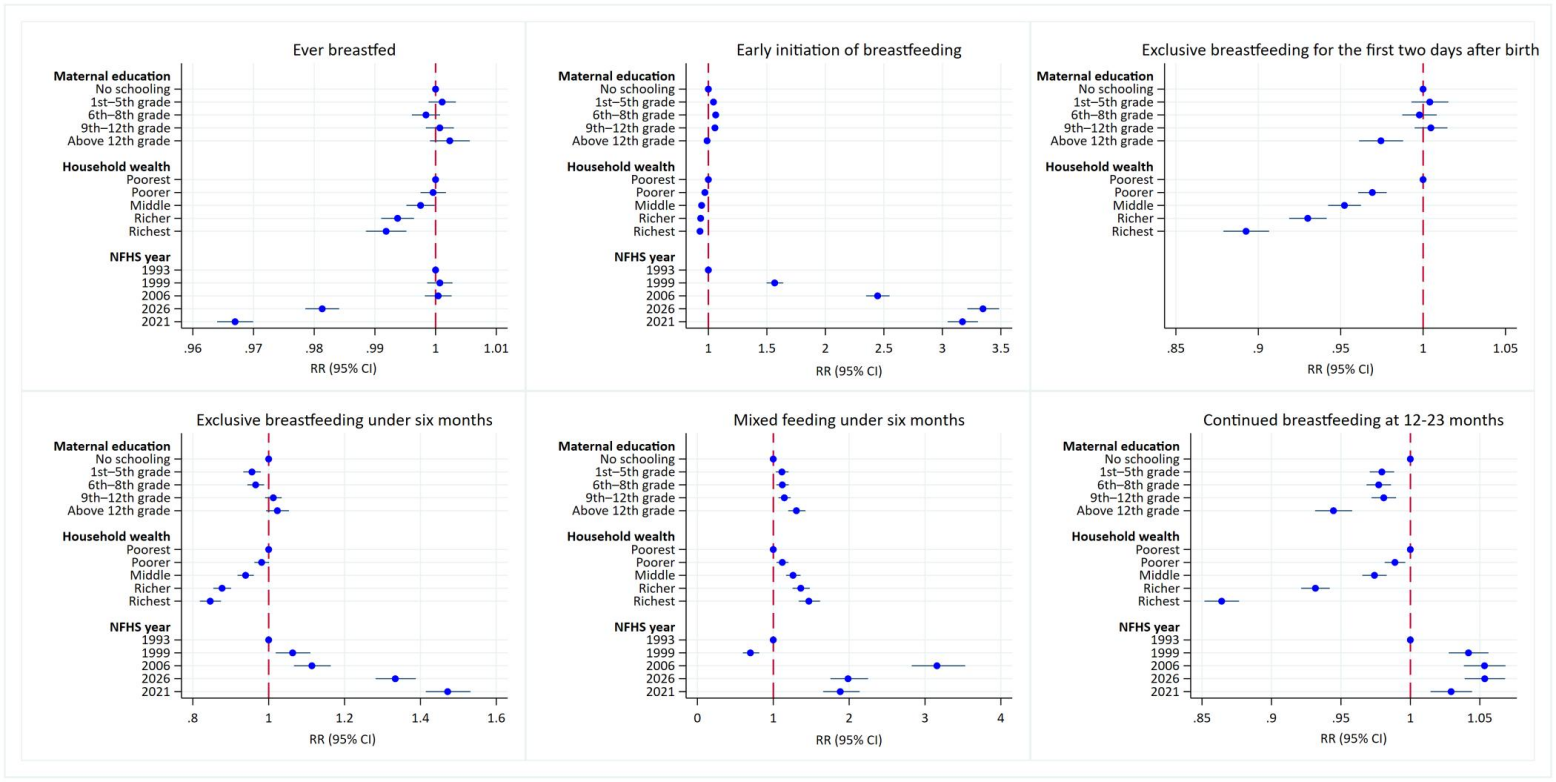
Role of maternal education and household wealth in the burden of breastfeeding indicators, adjusted for potential covariates including National Family Health Survey year. Details of the full model with estimated relative risks (RR) with 95% confidence intervals (CI) and p‐values are presented in the online supplement (Tables S5–S7). EvBF, ever breastfed; EIBF, early initiation of breastfeeding; EBF2D, exclusively breastfed for the first 2 days after birth; EBF, exclusive breastfeeding under 6 months; MixMF, mixed feeding under 6 months; CBF, continued breastfeeding 12–23 months.

## 4 Discussion

Utilizing five waves of nationally representative, cross‐sectional data, this study evaluated socioeconomic inequalities, as measured by maternal education and household wealth, in WHO‐defined breastfeeding indicators fundamental in surveillance and enhancement of infant nutrition. To quantify these disparities, absolute and relative inequality measures were employed in conjunction with the AAC to assess trends (1993–2021). The analysis demonstrates that disparities between the extremes of the socioeconomic distribution remain deeply embedded in breastfeeding practices, suggesting that progress has not been uniform across all population strata. These patterns in breastfeeding indicators were generally consistent across both measures of socioeconomic status (maternal education and household wealth). Consequently, it is imperative that policymakers design targeted interventions for both disadvantaged and privileged groups to eliminate persistent gaps and ensure the long‐term sustainability of optimal breastfeeding practices throughout India.

One of the most striking findings is the transition of EBF from a “pro‐poor” (progress was pro‐poor, meaning that improvements over time were progressively distributed, disproportionately benefiting the poorest sub‐populations relative to the richest) practice to one characterized by socioeconomic convergence. In 1993, children of mothers with no formal schooling and those from the poorest households were significantly more likely to be exclusively breastfed. By 2021, this gap had virtually closed. The shift toward a marginal advantage for the highest education group suggests that higher‐income and more educated mothers may have better access to lactation support and the flexibility to maintain EBF (Victora et al. 2016). The pattern we observe—an initial advantage among the least advantaged strata that erodes as the more advantaged catch up and overtake them—is one that the Inverse Equity Hypothesis would predict (Victora et al. 2000), under which the wealthiest and most educated strata adopt new public health interventions or guidelines first. We emphasize, however, that we did not test this hypothesis. Doing so would require data on the timing and reach of specific breastfeeding promotion activities across socioeconomic strata, which the NFHS does not collect. The consistency of our findings with the hypothesis is therefore suggestive, and alternative explanations, including differential change in employment patterns, in delivery practices, or in reporting behavior across strata, cannot be excluded on the evidence presented here.

Unlike EBF, EIBF exhibited a persistent “pro‐rich” (progress was pro‐rich, meaning that improvements over time were regressively distributed, disproportionately benefiting the richest sub‐populations relative to the poorest) and “pro‐educated” (where gains in EIBF were regressively concentrated among children of mothers with a higher education gradient (above 12th grade) compared to those with no formal education) bias, despite no statistically significant (*p* < 0.05) changes in the AI and RI across the study period (1993–2021). The AI between mothers with no schooling and those with more than a 12th‐grade education remained static over nearly three decades. Because EIBF relies on high‐quality postpartum care, healthcare utilization benefits were greatest for women attending ≥ 4 ANC visits and delivering in a facility (Table S5 in online supplement). One possible explanation for the persistent advantage held by more educated mothers is greater awareness of the benefits of early initiation, although the NFHS does not measure maternal knowledge of breastfeeding practice and we were therefore unable to examine this directly. A second possibility is that, although institutional deliveries have risen substantially in India, the quality of breastfeeding support provided within facilities varies by patient socioeconomic status (International Institute for Population Sciences IIPS and ICF 2021). Our data allow us to identify the place of delivery but not the content or quality of the postpartum support received, so this too remains untested. Both explanations are plausible interpretations of the pattern we observe rather than mechanisms we have demonstrated.

The nine percentage‐point increase in MixMF between 1993 and 2021 represents a significant public health challenge. The data show that this increase was most pronounced among the poorest wealth quintile and the least educated mothers. Aggressive marketing of breastmilk substitutes in India has been documented over this period (Kumari and Chandra 2025) and offers one candidate explanation for the rise in MixMF among the poorest children. We stress that this is a hypothesis and not a finding: we have no measure of exposure to marketing in the NFHS, and we did not test any association between marketing intensity and mixed milk feeding. Whether this increase stems from commercial promotion, perceptions of formula as an aspirational product (Jones et al. 2022), or rising maternal labor force participation requires purpose‐designed research. Overall, the rise in MixMF must be aggressively curbed, and national programs and policies should prioritize targeted, pro‐poor interventions that focus specifically on children from the poorest economic quintiles.

While this analysis provides a landmark 30‐year repeated cross‐sectional perspective based on a dataset heavily utilized by global health authorities, its interpretation is subject to certain constraints. First, information on breastfeeding indicators, including duration, was based on maternal recall. This reliance on recall may introduce reporting biases that could vary systematically by maternal education level, potentially confounding our inequality estimates. Consequently, a cautious interpretation of the observed socioeconomic disparities is warranted. Second, although missingness was modest, our complete‐case approach assumes that observations with missing data are missing at random conditional on the variables in the model. If the likelihood of a missing or non‐substantive response varies systematically with maternal education or household wealth, which we cannot rule out, the extreme‐group contrasts reported here could be biased in either direction, and this is most relevant for EvBF and EIBF in the 2016 and 2021 waves (Croft et al. 2023). Third, regarding household wealth, it should be noted that the indicators used to construct the wealth quintiles have evolved across successive NFHS waves, with the wealth index calculated separately for each survey. Because each index is standardized with a mean of zero and a standard deviation of one, specific scores and quintile classifications reflect relative economic status within a given survey wave (Rutstein and Staveteig 2014). Consequently, these temporal variations in the underlying index composition may influence the longitudinal comparability of breastfeeding trends. However, extensive methodological literature from the Demographic and Health Survey (DHS) Program (on which the NFHS design is based) demonstrates that the wealth index remains a highly robust proxy for stable relative social stratification over time (Filmer and Pritchett 2001; Rutstein and Johnson 2004). Because the index effectively ranks households within the contemporary socioeconomic hierarchy of each period (Rutstein 2008), evaluating trends across wealth quintiles captures the evolving distribution of breastfeeding indicators across structurally comparable segments of the population (Barros and Victora 2013; Gwatkin et al. 2007). Thus, the relative inequality gradients observed across these survey waves remain valid and highly informative for equity‐centered policy design. Fourth, a further limitation concerns the choice of inequality measure. Our estimates contrast only the extreme categories of maternal education and household wealth, describing the gap between the most and least advantaged strata rather than inequality across the whole socioeconomic distribution. Such contrasts are more sensitive to sampling variability than distribution‐wide measures, since they rest on the two smallest analytic subgroups, and they can shift for reasons unrelated to the underlying gradient: the share of mothers with no formal schooling fell substantially between 1993 and 2021, so the composition of that reference group changed over the study period. Finally, a primary limitation of assessing breastfeeding coverage inequality is that relative ratios and absolute proportion differences capture distinct mathematical properties, which can yield contradictory conclusions about disparity trends (Asada 2010; King et al. 2012). While relative metrics excel at scale‐invariant proportional comparisons, they can obscure severe absolute gaps. Conversely, absolute metrics quantify the tangible public health burden but remain highly sensitive to shifts in overall baseline prevalence (Atkinson and Brandolini 2010; King et al. 2012). Despite these constraints, combining both approaches provides a robust, multidimensional framework for mapping socioeconomic disparities in breastfeeding indicators across diverse populations (Asada 2010). Readers must carefully consider these structural limitations during interpretation, as relying on a single index can inadvertently present an incomplete portrait of healthcare equity (King et al. 2012; Niño‐Zarazúa et al. 2017).

## 5 Conclusions and Policy Implications

With the dissemination of select findings from NFHS‐6, global attention has been drawn to an abrupt downturn in EBF prevalence in 2024 (International Institute for Population Sciences IIPS 2026). This decline stands in stark contrast to the sustained national increase observed between 1993 and 2021 (Rai et al. 2026). Notably, diminished EBF coverage from 2021 to 2024 was recorded across over 20 states and union territories (see Table S8 in the online supplement and Figure 4). Paradoxically, while indicators for various maternal healthcare services have shown an upward trend (International Institute for Population Sciences IIPS 2026), the factors driving the recent decline in EBF demand rigorous investigation, particularly regarding the extent of heterogeneity across socioeconomic strata in India. Consistent with prior literature in India (Debnath et al. 2021), investigating whether maternal reluctance contributes to this decline is a critical pathway that can be rigorously evaluated once the NFHS‐6 data are made available to the research community. To strengthen child health in India, priority must be given to isolating the primary drivers of the EBF decline and formulating targeted interventions through the revitalization of the MAA flagship program (Ministry of Health and Family Welfare 2016).

**Figure 4:**
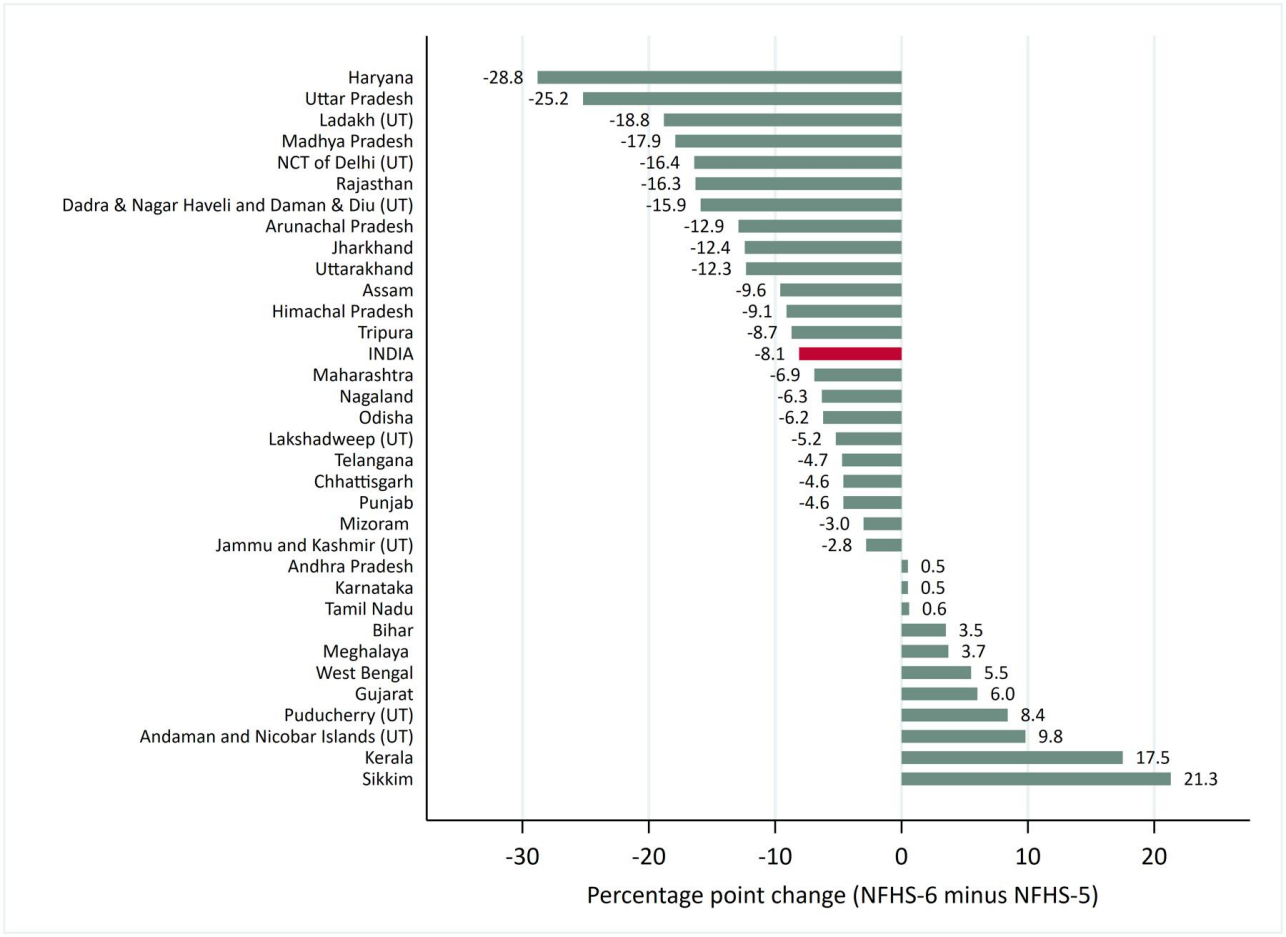
Percentage point change (between 2019–2021 and 2023–2024) in exclusive breastfeeding prevalence in select states and union territories (UTs). Prevalence estimates for Chandigarh (UT) and Goa were not available in the NFHS‐6 fact sheets as the denominators had fewer than 25 unweighted cases, whereas estimates for the state of Manipur were not available in the NFHS‐6 fact sheet (2023–2024) for comparison with NFHS‐5 (2019–2021).

Analysis of socioeconomic inequalities spanning NFHS‐1 through NFHS‐5 suggests that while most breastfeeding indicators improved across all socioeconomic strata, these gains did not necessarily narrow the equity gap in quality‐dependent practices such as EIBF. Consequently, enhancing EIBF coverage requires more robust postpartum care systems that specifically target marginalized demographics, such as women with no formal education or those within the lowest wealth quintile, which in turn serves as a critical prerequisite for expanding overall EBF coverage. Furthermore, efforts to expand EBF coverage will necessitate sustained strategies to reduce the prevalence of MixMF, even among disadvantaged populations, given that MixMF rates have consistently risen between 1993 and 2021. A potentially effective strategy involves the active monitoring of EBF practices by community‐level health workers across both rural and urban India, thereby systematically addressing localized barriers to EBF within the operational framework of the national MAA program (Ministry of Health and Family Welfare 2016). Failure to address these underlying drivers may jeopardize India's ability to meet the 2030 Global Nutrition Target (World Health Organization 2025).

## Author Contributions

R.K.R. and R.K. conceptualized the study. R.K.R. performed the data analysis and led the writing, revision, and finalization of the manuscript. J.W.D.N., S.B., R.K., and S.V.S. reviewed and provided feedback on manuscript drafts and revisions. All authors had full access to the entire datasets used in the study and collectively assumed responsibility for the decision to submit for publication.

## Conflicts of Interest

The authors declare no conflicts of interest.

## Supporting information


Supporting File

## Data Availability

Data from the National Family Health Surveys, as detailed in the manuscript, can be obtained by submitting a request through the DHS Program's official website: https://dhsprogram.com/.
