# Supplementary material for: Three Decades of Socioeconomic Inequality in India's Breastfeeding Indicators, 1993–2021
Source: Matern Child Nutr. 2026 Oct 15;22(4):e70258. doi: 10.1111/mcn.70258 (PMC13670010; doi:10.1111/mcn.70258)
Supplement: Supplementary file 1 [file MCN-22-e70258-s001.pdf]

Three decades of socioeconomic inequality in India's breastfeeding indicators, 1993–2021

Rajesh Kumar Rai [1,2,3,4,5,*], Jan-Walter De Neve [1,6], Sabri Bromage [7,8], Rockli Kim [9,10,*], S V Subramanian [11,12]

[1]Division of Global Health Management and Policy, School of Public Health, San Diego State University, San Diego, California, USA; [2]Birbhum Population Project, Society for Health and Demographic Surveillance, Suri, West Bengal, India; [3]ImpactLoop LTD, London, UK; [4]Human Nutrition Unit, Institute of Nutrition, Mahidol University, Phutthamonthon, Nakhon Pathom, Thailand; [5]Department of Global Health and Population, Harvard T H Chan School of Public Health, Boston, Massachusetts, USA; [6]Heidelberg Institute of Global Health, Faculty of Medicine and University Hospital, University of Heidelberg, Heidelberg, Germany; [7]Community Nutrition Unit, Institute of Nutrition, Mahidol University, Phutthamonthon, Nakhon Pathom, Thailand; [8]Department of Nutrition, Harvard T H Chan School of Public Health, Boston, Massachusetts, USA; [9]Division of Health Policy and Management, College of Health Science, Korea University, Seoul, South Korea; [10]Interdisciplinary Program in Precision Public Health, Department of Public Health Sciences, Graduate School of Korea University, Seoul, South Korea; [11]Harvard Center for Population and Development Studies, Cambridge, Massachusetts, USA; [12]Department of Social and Behavioral Sciences, Harvard T H Chan School of Public Health, Boston, Massachusetts, USA.

* *Joint corresponding author*

Correspondence: Rajesh Kumar Rai (rrai@sdsu.edu, rajesh.iips28@gmail.com)
Rockli Kim (rocklikim@korea.ac.kr)

Online supplement

Table S1. Prevalence of breastfeeding indicators by maternal education, NFHS (1993-2021).

	1993<br>% (95% CI)	1999<br>% (95% CI)	2006<br>% (95% CI)	2016<br>% (95% CI)	2021<br>% (95% CI)
			**Ever breastfed**		
No schooling	99.3 (99.1, 99.4)	99.1 (98.9, 99.3)	99.4 (99.1, 99.5)	96.8 (96.5, 97.2)	94.5 (94.0, 95.0)
1st–5th grade	98.7 (98.0, 99.1)	99.1 (98.6, 99.4)	98.8 (98.1, 99.3)	97.4 (96.9, 97.8)	95.4 (94.8, 96.0)
6th–8th grade	98.1 (97.3, 98.6)	99.0 (98.4, 99.4)	99.0 (98.5, 99.4)	96.8 (96.4, 97.2)	95.3 (94.8, 95.7)
9th–12th grade	97.9 (97.2, 98.5)	98.4 (97.8, 98.9)	98.7 (98.1, 99.1)	97.1 (96.8, 97.4)	95.6 (95.2, 95.9)
Above 12th grade	98.4 (96.9, 99.1)	97.7 (95.8, 98.8)	98.7 (97.6, 99.2)	96.5 (95.9, 97.1)	96.1 (95.7, 96.5)
			**Early initiation of breastfeeding**		
No schooling	8.7 (7.9, 9.5)	13.1 (11.9, 14.4)	16.1 (15.0, 17.3)	37.2 (36.3, 38.2)	36.7 (35.7, 37.8)
1st–5th grade	10.1 (8.8, 11.7)	19.4 (17.6, 21.4)	23.7 (21.7, 25.9)	41.5 (40.1, 42.9)	40.7 (39.3, 42.1)
6th–8th grade	11.2 (9.6, 13.0)	21.4 (19.2, 23.6)	29.6 (27.3, 32.0)	44.5 (43.3, 45.7)	40.9 (39.8, 42.0)
9th–12th grade	11.7 (10.2, 13.3)	23.0 (20.9, 25.2)	34.2 (32.1, 36.3)	46.2 (45.2, 47.1)	44.0 (43.2, 44.9)
Above 12th grade	13.2 (10.3, 16.9)	20.6 (17.7, 23.7)	34.8 (31.4, 38.4)	40.7 (39.2, 42.2)	41.0 (39.5, 42.4)
			**Exclusively breastfed for the first two days after birth**		
No schooling	rina	rina	rina	rina	81.3 (80.5, 82.2)
1st–5th grade	rina	rina	rina	rina	82.6 (81.6, 83.6)
6th–8th grade	rina	rina	rina	rina	81.2 (80.4, 82.0)
9th–12th grade	rina	rina	rina	rina	81.3 (80.6, 81.9)
Above 12th grade	rina	rina	rina	rina	76.5 (75.4, 77.5)
			**Exclusive breastfeeding under six months**		
No schooling	50.5 (48.4, 52.5)	52.7 (49.8, 55.7)	47.9 (44.8, 50.9)	53.8 (52.2, 55.5)	62.3 (60.4, 64.3)
1st–5th grade	39.3 (34.8, 44.0)	43.9 (38.6, 49.4)	49.3 (44.5, 54.1)	53.6 (51.1, 56.2)	61.6 (58.9, 64.2)
6th–8th grade	30.4 (26.0, 35.2)	40.2 (35.0, 45.6)	42.5 (38.0, 47.3)	52.1 (49.9, 54.3)	64.9 (62.9, 66.9)
9th–12th grade	21.3 (17.4, 25.7)	39.1 (34.5, 43.8)	44.1 (39.9, 48.5)	57.7 (55.9, 59.5)	64.5 (62.8, 66.1)
Above 12th grade	21.8 (12.8, 34.6)	21.3 (14.9, 29.6)	41.4 (34.5, 48.6)	54.7 (51.5, 57.8)	63.7 (61.5, 65.9)
			**Mixed feeding under six months**		
No schooling	2.4 (1.9, 3.1)	2.4 (1.7, 3.4)	15.7 (13.6, 17.9)	13.4 (12.3, 14.6)	13.9 (12.5, 15.4)
1st–5th grade	6.1 (4.3, 8.6)	5.4 (3.5, 8.1)	19.8 (16.4, 23.7)	14.1 (12.5, 15.9)	13.4 (11.7, 15.4)
6th–8th grade	9.3 (6.9, 12.3)	6.5 (4.2, 9.9)	21.9 (18.2, 26.1)	16.4 (14.6, 18.5)	12.8 (11.5, 14.4)
9th–12th grade	14.0 (10.8, 18.1)	6.7 (4.7, 9.5)	26.3 (23.0, 29.9)	15.4 (14.2, 16.8)	13.8 (12.7, 14.9)
Above 12th grade	17.9 (11.3, 27.0)	16.6 (10.7, 24.7)	32.7 (26.2, 39.9)	18.6 (16.6, 20.8)	16.7 (15.0, 18.5)
			**Continued breastfeeding 12-23 months**		
No schooling	85.9 (84.8, 86.9)	87.6 (86.6, 88.6)	83.4 (81.9, 84.8)	79.7 (78.8, 80.6)	78.0 (76.8, 79.2)
1st–5th grade	75.9 (73.2, 78.4)	80.5 (77.7, 83.1)	80.8 (77.9, 83.4)	78.7 (77.4, 80.1)	77.9 (76.3, 79.5)
6th–8th grade	71.0 (68.2, 73.7)	76.0 (72.8, 79.0)	78.6 (75.8, 81.2)	76.2 (74.7, 77.6)	77.3 (76.0, 78.6)
9th–12th grade	60.7 (57.3, 64.0)	69.3 (66.4, 72.1)	70.6 (67.9, 73.2)	72.9 (71.7, 74.0)	75.2 (74.2, 76.2)
Above 12th grade	44.9 (38.6, 51.5)	49.9 (44.4, 55.5)	58.2 (53.4, 62.9)	66.9 (64.7, 69.0)	69.6 (68.0, 71.2)

CI: confidence interval, NFHS: National Family Health Survey, rina: required information not available

Table S2. Prevalence of breastfeeding indicators by household wealth, NFHS (1993-2021).

	1993<br>% (95% CI)	1999<br>% (95% CI)	2006<br>% (95% CI)	2016<br>% (95% CI)	2021<br>% (95% CI)
			**Ever breastfed**		
Poorest	99.3 (98.9, 99.5)	99.2 (98.7, 99.4)	99.5 (99.1, 99.7)	97.3 (97.0, 97.6)	94.9 (94.5, 95.3)
Poorer	99.4 (99.0, 99.6)	99.3 (98.9, 99.5)	99.3 (98.9, 99.6)	97.2 (96.9, 97.5)	95.2 (94.7, 95.6)
Middle	99.0 (98.5, 99.3)	99.1 (98.7, 99.4)	99.0 (98.5, 99.3)	97.0 (96.7, 97.4)	95.6 (95.1, 96.0)
Richer	98.4 (98.0, 98.8)	98.8 (98.3, 99.1)	99.1 (98.6, 99.4)	96.6 (96.0, 97.0)	95.5 (95.0, 95.9)
Richest	98.2 (97.7, 98.6)	98.3 (97.6, 98.8)	98.1 (97.4, 98.7)	96.3 (95.6, 96.9)	96.0 (95.5, 96.5)
			**Early initiation of breastfeeding**		
Poorest	9.1 (7.9, 10.4)	14.8 (13.3, 16.5)	17.0 (15.3, 18.7)	39.9 (38.9, 40.8)	39.1 (38.1, 40.0)
Poorer	8.6 (7.6, 9.7)	15.4 (13.6, 17.4)	20.0 (18.4, 21.8)	43.0 (42.0, 44.0)	38.9 (37.8, 39.9)
Middle	9.4 (8.1, 10.8)	17.7 (16.3, 19.1)	25.3 (23.4, 27.3)	43.9 (42.7, 45.1)	42.3 (41.2, 43.5)
Richer	9.7 (8.6, 10.9)	19.2 (17.5, 21.0)	29.1 (27.1, 31.2)	44.0 (42.7, 45.2)	44.0 (42.7, 45.3)
Richest	11.7 (10.3, 13.3)	18.4 (16.7, 20.2)	30.8 (28.6, 33.1)	40.1 (38.6, 41.6)	43.0 (41.6, 44.4)
			**Exclusively breastfed for the first two days after birth**		
Poorest	rina	rina	rina	rina	83.6 (82.9, 84.3)
Poorer	rina	rina	rina	rina	81.8 (81.0, 82.6)
Middle	rina	rina	rina	rina	81.2 (80.3, 82.0)
Richer	rina	rina	rina	rina	79.1 (78.1, 80.1)
Richest	rina	rina	rina	rina	75.3 (74.1, 76.5)
			**Exclusive breastfeeding under six months**		
Poorest	57.4 (53.9, 60.9)	57.5 (53.0, 61.8)	53.1 (49.2, 57.0)	55.7 (54.0, 57.4)	64.7 (63.0, 66.4)
Poorer	52.1 (48.4, 55.8)	52.3 (48.1, 56.4)	51.0 (46.9, 55.1)	56.6 (54.7, 58.4)	64.7 (62.9, 66.5)
Middle	43.3 (39.6, 47.0)	48.1 (43.7, 52.5)	43.0 (38.8, 47.2)	54.2 (52.1, 56.4)	65.2 (63.3, 67.1)
Richer	34.6 (31.3, 38.2)	38.9 (35.2, 42.8)	41.8 (37.5, 46.2)	52.9 (50.4, 55.4)	61.2 (58.8, 63.5)
Richest	18.7 (15.4, 22.6)	28.7 (24.6, 33.1)	36.0 (31.5, 40.8)	53.6 (50.8, 56.3)	61.9 (59.3, 64.4)
			**Mixed feeding under six months**		
Poorest	2.4 (1.6, 3.6)	3.3 (2.2, 5.1)	12.5 (10.1, 15.4)	12.9 (11.8, 14.1)	11.9 (10.8, 13.1)
Poorer	2.4 (1.5, 3.7)	2.8 (1.8, 4.4)	18.1 (15.2, 21.4)	13.5 (12.3, 14.8)	12.6 (11.4, 13.9)
Middle	4.2 (3.1, 5.7)	4.5 (3.1, 6.5)	19.0 (16.0, 22.3)	15.5 (14.0, 17.0)	14.5 (13.1, 16.0)
Richer	6.0 (4.6, 7.9)	5.1 (3.6, 7.1)	25.6 (22.0, 29.6)	17.0 (15.2, 19.1)	15.7 (14.2, 17.4)
Richest	14.1 (11.4, 17.3)	8.4 (6.3, 11.0)	31.2 (27.2, 35.6)	19.9 (17.8, 22.2)	17.5 (15.5, 19.7)
			**Continued breastfeeding 12-23 months**		
Poorest	89.9 (88.2, 91.4)	90.8 (89.1, 92.2)	86.2 (84.1, 87.9)	83.0 (82.2, 83.9)	81.4 (80.4, 82.4)
Poorer	88.0 (86.4, 89.5)	87.5 (85.6, 89.2)	84.2 (82.0, 86.2)	79.3 (78.2, 80.4)	78.3 (77.2, 79.4)
Middle	81.3 (79.0, 83.3)	81.7 (79.4, 83.8)	80.0 (77.6, 82.2)	74.9 (73.6, 76.1)	75.2 (73.8, 76.5)
Richer	72.8 (70.6, 74.9)	75.0 (72.3, 77.5)	72.9 (70.1, 75.5)	69.6 (68.1, 71.1)	71.2 (69.7, 72.6)
Richest	57.4 (54.6, 60.2)	63.2 (60.2, 66.1)	63.3 (60.4, 66.0)	65.6 (63.5, 67.6)	68.2 (66.5, 69.9)

CI: confidence interval, NFHS: National Family Health Survey, rina: required information not available

Table S3. Annual absolute changes in breastfeeding indicators by maternal education: NFHS (1993–1999, 1999–2006, 2006–2016, 2016–2021, and 1993-2021).

	1993-1999	1999-2006	2006-2016	2016-2021	1993-2021
			**Ever breastfed**		
No schooling	-0.03	0.04	-0.26	-0.46	-0.16
1st–5th grade	0.07	-0.04	-0.14	-0.40	-0.11
6th–8th grade	0.15	<0.01	-0.22	-0.30	-0.09
9th–12th grade	0.08	0.04	-0.16	-0.30	-0.08
Above 12th grade	-0.12	0.14	-0.22	-0.08	-0.08
			**Early initiation of breastfeeding**		
No schooling	0.73	0.43	2.11	-0.10	0.93
1st–5th grade	1.55	0.61	1.78	-0.16	1.02
6th–8th grade	1.70	1.17	1.49	-0.72	0.99
9th–12th grade	1.88	1.60	1.20	-0.44	1.08
Above 12th grade	1.23	2.03	0.59	0.06	0.93
			**Exclusive breastfeeding under six months**		
No schooling	0.37	-0.69	0.59	1.70	0.39
1st–5th grade	0.77	0.77	0.43	1.60	0.74
6th–8th grade	1.63	0.33	0.96	2.56	1.15
9th–12th grade	2.97	0.71	1.36	1.36	1.44
Above 12th grade	-0.08	2.87	1.33	1.80	1.40
			**Mixed feeding under six months**		
No schooling	<0.01	1.90	-0.23	0.10	0.38
1st–5th grade	-0.12	2.06	-0.57	-0.14	0.24
6th–8th grade	-0.47	2.20	-0.55	-0.72	0.12
9th–12th grade	-1.22	2.80	-1.09	-0.32	-0.01
Above 12th grade	-0.22	2.30	-1.41	-0.38	-0.04
			**Continued breastfeeding 12-23 months**		
No schooling	0.28	-0.60	-0.37	-0.34	-0.26
1st–5th grade	0.77	0.04	-0.21	-0.16	0.07
6th–8th grade	0.83	0.37	-0.24	0.22	0.21
9th–12th grade	1.43	0.19	0.23	0.46	0.48
Above 12th grade	0.83	1.19	0.87	0.54	0.82

NFHS: National Family Health Survey

Table S4. Annual absolute changes in breastfeeding indicators by household wealth: NFHS (1993–1999, 1999–2006, 2006–2016, 2016–2021, and 1993-2021).

	1993-1999	1999-2006	2006-2016	2016-2021	1993-2021
			**Ever breastfed**		
Poorest	-0.02	0.04	-0.22	-0.48	-0.15
Poorer	-0.02	<0.01	-0.21	-0.40	-0.14
Middle	0.02	-0.01	-0.20	-0.28	-0.11
Richer	0.07	0.04	-0.25	-0.22	-0.10
Richest	0.02	-0.03	-0.18	-0.06	-0.07
			**Early initiation of breastfeeding**		
Poorest	0.95	0.31	2.29	-0.16	1.00
Poorer	1.13	0.66	2.30	-0.82	1.01
Middle	1.38	1.09	1.86	-0.32	1.10
Richer	1.58	1.41	1.49	0.00	1.14
Richest	1.12	1.77	0.93	0.58	1.04
			**Exclusive breastfeeding under six months**		
Poorest	0.02	-0.63	0.26	1.80	0.24
Poorer	0.03	-0.19	0.56	1.62	0.42
Middle	0.80	-0.73	1.12	2.20	0.73
Richer	0.72	0.41	1.11	1.66	0.89
Richest	1.67	1.04	1.76	1.66	1.44
			**Mixed feeding under six months**		
Poorest	0.15	1.31	0.04	-0.20	0.32
Poorer	0.07	2.19	-0.46	-0.18	0.34
Middle	0.05	2.07	-0.35	-0.20	0.34
Richer	-0.15	2.93	-0.86	-0.26	0.32
Richest	-0.95	3.26	-1.13	-0.48	0.11
			**Continued breastfeeding 12-23 months**		
Poorest	0.15	-0.66	-0.32	-0.32	-0.28
Poorer	-0.08	-0.47	-0.49	-0.20	-0.32
Middle	0.07	-0.24	-0.51	0.06	-0.20
Richer	0.37	-0.30	-0.33	0.32	-0.05
Richest	0.97	0.01	0.23	0.52	0.36

NFHS: National Family Health Survey

Table S5. Association of maternal education and household wealth with ever breastfeeding and early initiation of breastfeeding in India.

	Ever breastfeeding			Early initiation of breastfeeding		
	Sample (241,868) [†]	% (95% CI)	RR (95% CI) *p*	Sample (242,050) [†]	% (95% CI)	RR (95% CI) *p*
Maternal education						
No schooling	73,321	97.7 (97.5, 97.9)	1.000	73,381	25.7 (25.2, 26.1)	1.00
1st–5th grade	31,729	97.5 (97.2, 97.8)	1.001 (0.999, 1.003) 0.343	31,749	34.7 (33.8, 35.5)	1.04 (1.03, 1.06) <0.001
6th–8th grade	40,656	97.0 (96.7, 97.2)	0.998 (0.996,1.001) 0.181	40,681	38.6 (37.8, 39.4)	1.06 (1.04, 1.08) <0.001
9th–12th grade	69,126	96.9 (96.7, 97.2)	1.001 (0.998, 1.003) 0.556	69,188	42.4 (41.8, 43.0)	1.06 (1.04, 1.07) <0.001
Above 12th grade	27,036	96.7 (96.4, 97.1)	1.002 (0.999, 1.006) 0.160	27,051	39.5 (38.4, 40.5)	0.99 (0.97, 1.01) 0.336
Household wealth						
Poorest	58,054	97.3 (97.1, 97.5)	1.000	58,094	33.1 (32.5, 33.7)	1.00
Poorer	53,383	97.4 (97.2, 97.6)	1.000 (0.998, 1.002) 0.707	53,429	34.1 (33.5, 34.8)	0.97 (0.96, 0.99) <0.001
Middle	48,473	97.4 (97.2, 97.6)	0.998 (0.995, 1.000) 0.039	48,506	36.4 (35.7, 37.2)	0.94 (0.93, 0.96) <0.001
Richer	44,683	97.0 (96.8, 97.3)	0.994 (0.991, 0.996) <0.001	44,714	37.2 (36.4, 38.0)	0.93 (0.92, 0.95) <0.001
Richest	37,275	96.9 (96.6, 97.2)	0.992 (0.989, 0.995) <0.001	37,307	35.9 (35.0, 36.8)	0.93 (0.91, 0.95) <0.001
Maternal age (year)						
15-19	16,102	97.8 (97.5, 98.2)	1.000	16,109	30.4 (29.3, 31.5)	1.00
20-24	92,710	97.3 (97.2, 97.5)	0.998 (0.995, 1.000) 0.107	92,755	35.5 (35.0, 36.0)	1.01 (0.99, 1.04) 0.248
25-29	82,153	97.1 (96.9, 97.3)	0.996 (0.993, 0.999) 0.006	82,215	36.5 (36.0, 37.1)	1.01 (0.99, 1.04) 0.294
30-34	35,148	97.1 (96.9, 97.4)	0.994 (0.991, 0.997) 0.001	35,193	35.2 (34.3, 36.0)	1.00 (0.97, 1.03) 0.961
35-39	12,331	96.5 (95.9, 97.1)	0.988 (0.984, 0.993) <0.001	12,349	32.0 (30.6, 33.3)	0.99 (0.96, 1.02) 0.427
40-44	2,782	95.7 (94.2, 97.1)	0.985 (0.977, 0.992) <0.001	2,786	29.9 (26.9, 32.8)	0.99 (0.94, 1.04) 0.651
45-49	642	96.3 (94.1, 98.6)	0.975 (0.959, 0.992) 0.005	643	31.0 (24.2, 37.7)	1.01 (0.91, 1.11) 0.905
Mass media exposure						
No	79,298	97.4 (97.2, 97.5)	1.000	79,367	28.6 (28.1, 29.1)	1.00
Yes	162,570	97.2 (97.0, 97.3)	1.003 (1.001, 1.005) 0.002	162,683	38.6 (38.2, 39.0)	1.06 (1.05, 1.08) <0.001
Number of ANC visits						
<4	126,237	97.1 (96.9, 97.2)	1.000	126,347	28.1 (27.7, 28.5)	1.00
≥4	115,631	97.4 (97.2, 97.5)	1.012 (1.010, 1.013) <0.001	115,703	42.8 (42.3, 43.2)	1.17 (1.16, 1.18) <0.001
Place of delivery						
Home/others	67,951	98.2 (98.1, 98.4)	1.000	67,989	20.6 (20.1, 21.0)	1.00
Healthcare facility	173,917	96.9 (96.7, 97.0)	0.997 (0.995, 0.999) 0.002	174,061	40.7 (40.3, 41.1)	1.12 (1.11, 1.14) <0.001
Birth order						
First	86,426	96.8 (96.6, 97.0)	1.000	86,476	36.1 (35.5, 36.6)	1.00
Second	75,146	97.4 (97.2, 97.5)	1.007 (1.005, 1.009) <0.001	75,201	38.5 (37.9, 39.1)	1.09 (1.08, 1.10) <0.001
Third or higher	80,296	97.5 (97.4, 97.7)	1.008 (1.006, 1.010) <0.001	80,373	31.0 (30.5, 31.5)	1.12 (1.10, 1.14) <0.001
Child age (in months)	241,868	[§]	1.000 (1.000, 1.000) <0.001	242,050	[§]	1.00 (1.00, 1.00) 0.088

Sex of child						
Male	125,773	97.1 (97.0, 97.3)	1.000	125,869	35.4 (34.9, 35.8)	1.00
Female	116,095	97.3 (97.2, 97.5)	1.002 (1.000, 1.003) 0.012	116,181	35.1 (34.6, 35.5)	1.01 (1.00, 1.02) 0.137
Household size	241,868	§	1.001 (1.000, 1.001) <0.001	242,050	§	0.99 (0.99, 1.00) <0.001
Social group						
Scheduled castes	153,768	96.8 (96.7, 96.9)	1.000	153,863	39.4 (39.0, 39.7)	1.00
Scheduled tribes	37,720	97.8 (97.5, 98.1)	1.001 (0.998, 1.003) 0.561	37,775	38.5 (37.5, 39.4)	1.14 (1.12, 1.16) <0.001
Others	50,380	98.3 (98.1, 98.5)	0.996 (0.993, 0.998) <0.001	50,412	21.2 (20.6, 21.8)	0.97 (0.95, 0.99) 0.007
Religion						
Hinduism	177,024	97.4 (97.2, 97.5)	1.000	177,127	34.7 (34.3, 35.0)	1.00
Islam	35,955	96.7 (96.4, 97.0)	0.993 (0.991, 0.995) <0.001	35,981	35.7 (34.8, 36.5)	1.01 (0.99, 1.02) 0.329
Others	28,889	96.9 (96.4, 97.3)	0.991 (0.988, 0.994) <0.001	28,942	42.6 (41.2, 44.0)	1.04 (1.02, 1.05) <0.001
Locality of residence						
Urban	57,499	96.9 (96.6, 97.1)	1.000	57,542	38.2 (37.4, 38.9)	1.00
Rural	184,369	97.4 (97.2, 97.5)	0.999 (0.998, 1.001) 0.559	184,508	34.2 (33.8, 34.5)	1.02 (1.01, 1.03) 0.003
Region of residence						
North	46,851	97.2 (96.9, 97.4)	1.000	46,875	31.9 (31.2, 32.5)	1.00
South	29,453	96.6 (96.3, 97.0)	0.996 (0.994, 0.999) 0.006	29,489	45.3 (44.5, 46.2)	1.28 (1.26, 1.30) <0.001
East	47,002	97.3 (97.0, 97.5)	1.005 (1.002, 1.007) <0.001	47,042	35.6 (35.0, 36.3)	1.06 (1.04, 1.08) <0.001
West	20,921	98.1 (97.7, 98.4)	1.008 (1.006, 1.011) <0.001	20,941	43.7 (42.6, 44.9)	1.24 (1.21, 1.26) <0.001
Central	63,181	97.2 (97.1, 97.4)	1.003 (1.000, 1.005) 0.017	63,197	23.8 (23.3, 24.2)	0.77 (0.76, 0.78) <0.001
Northeast	34,460	97.0 (96.7, 97.4)	1.002 (0.999, 1.005) 0.106	34,506	52.0 (51.1, 53.0)	1.49 (1.46, 1.51) <0.001
NFHS year[‡]						
1993	23,299	98.9 (98.7, 99.1)	1.000	23,319	9.6 (9.0, 10.1)	1.00
1999	19,045	99.0 (98.8, 99.2)	1.001 (0.999, 1.003) 0.511	19,051	17.0 (16.3, 17.8)	1.57 (1.50, 1.64) <0.001
2006	18,455	99.2 (99.0, 99.4)	1.000 (0.998, 1.003) 0.701	18,458	23.6 (22.7, 24.5)	2.45 (2.35, 2.55) <0.001
2016	93,311	97.3 (97.1, 97.5)	0.981 (0.979, 0.984) <0.001	93,368	42.3 (41.8, 42.9)	3.35 (3.21, 3.49) <0.001
2021	87,758	95.9 (95.7, 96.1)	0.967 (0.964, 0.970) <0.001	87,854	41.4 (40.9, 42.0)	3.17 (3.05, 3.30) <0.001

Note: Analysis based on pooled data from the five waves of the National Family Health Survey, 1993–2021.

† All samples are unweighted.

‡ Compared with Figure S1, the cumulative sample sizes across survey rounds may vary slightly because cases with missing values on key independent variables were excluded from the analysis.

§ Percentages were not calculated because this is a continuous (count) variable.

ANC: antenatal care, CI: confidence interval, NFHS: National Family Health Survey, RR: relative risk

Table S6. Association of maternal education and household wealth with exclusive breastfeeding for the first two days after birth and exclusive breastfeeding under six months in India.

	**Exclusive breastfeeding for the first two days after birth**			**Exclusive breastfeeding under six months**		
	Sample (87,854) †	% (95% CI)	RR (95% CI) *p*	Sample (60,889) †	% (95% CI)	RR (95% CI) *p*
Maternal education						
No schooling	17,174	81.9 (81.1, 82.6)	1.00	18,469	54.1 (53.1, 55.1)	1.00
1st–5th grade	10,259	83.1 (82.1, 84.0)	1.00 (0.99, 1.02) 0.476	7,979	53.6 (51.8, 55.3)	0.96 (0.93, 0.98) <0.001
6th–8th grade	15,740	81.8 (80.9, 82.6)	1.00 (0.99, 1.01) 0.675	10,139	54.5 (52.9, 56.1)	0.97 (0.94, 0.99) 0.003
9th–12th grade	31,084	81.7 (81.1, 82.3)	1.00 (0.99, 1.01) 0.348	17,378	58.0 (56.8, 59.3)	1.01 (0.99, 1.03) 0.275
Above 12th grade	13,597	76.7 (75.7, 77.7)	0.97 (0.96, 0.99) <0.001	6,924	57.7 (55.7, 59.7)	1.02 (0.99, 1.05) 0.131
Household wealth						
Poorest	23,366	84.1 (83.5, 84.8)	1.00	14,905	59.2 (58.1, 60.4)	1.00
Poorer	20,305	82.4 (81.6, 83.1)	0.97 (0.96, 0.98) <0.001	13,764	58.3 (57.0, 59.6)	0.98 (0.96, 1.00) 0.063
Middle	17,254	81.6 (80.9, 82.4)	0.95 (0.94, 0.96) <0.001	12,137	55.7 (54.3, 57.1)	0.94 (0.92, 0.96) <0.001
Richer	15,009	79.5 (78.6, 80.5)	0.93 (0.92, 0.94) <0.001	11,023	52.0 (50.4, 53.6)	0.88 (0.85, 0.90) <0.001
Richest	11,920	75.5 (74.4, 76.6)	0.89 (0.88, 0.91) <0.001	9,060	50.1 (48.3, 51.9)	0.85 (0.82, 0.87) <0.001
Maternal age (year)						
15-19	4,292	82.1 (80.6, 83.6)	1.00	5,680	56.8 (54.8, 58.8)	1.00
20-24	32,900	81.2 (80.6, 81.7)	1.00 (0.98, 1.01) 0.652	24,832	56.2 (55.3, 57.2)	1.00 (0.98, 1.02) 0.935
25-29	31,672	81.6 (81.0, 82.2)	0.99 (0.97, 1.00) 0.077	19,366	55.1 (54.0, 56.2)	1.01 (0.98, 1.04) 0.482
30-34	13,345	79.4 (78.4, 80.5)	0.96 (0.95, 0.98) <0.001	7,660	55.1 (53.2, 57.0)	1.04 (1.01, 1.07) 0.020
35-39	4,544	79.1 (77.4, 80.9)	0.94 (0.92, 0.96) <0.001	2,660	52.1 (48.9, 55.3)	0.99 (0.95, 1.04) 0.813
40-44	903	81.5 (77.7, 85.2)	0.96 (0.92, 0.99) 0.012	559	52.4 (45.6, 59.2)	1.02 (0.95, 1.11) 0.574
45-49	198	78.2 (69.0, 87.5)	0.93 (0.87, 1.00) 0.058	132	49.2 (35.2, 63.2)	0.98 (0.83, 1.16) 0.808
Mass media exposure						
No	27,501	82.0 (81.4, 82.6)	1.00	20,489	55.3 (54.3, 56.3)	1.00
Yes	60,353	80.6 (80.1, 81.0)	1.01 (1.00, 1.01) 0.088	40,400	55.8 (55.0, 56.6)	1.03 (1.01, 1.04) 0.003
Number of ANC visits						
<4	37,692	79.5 (79.0, 80.1)	1.00	32,092	53.5 (52.6, 54.3)	1.00
≥4	50,162	82.1 (81.6, 82.6)	1.04 (1.03, 1.05) <0.001	28,797	58.0 (57.0, 58.9)	1.03 (1.01, 1.04) <0.001
Place of delivery						
Home/others	10,233	76.6 (75.4, 77.8)	1.00	17,331	51.0 (49.9, 52.1)	1.00
Healthcare facility	77,621	81.5 (81.1, 81.9)	1.07 (1.05, 1.08) <0.001	43,558	57.4 (56.6, 58.2)	0.97 (0.96, 0.99) 0.011
Birth order						
First	33,955	78.1 (77.5, 78.7)	1.00	21,893	60.4 (59.4, 61.5)	1.00
Second	29,188	82.6 (82.0, 83.3)	1.06 (1.05, 1.07) <0.001	18,628	52.0 (50.8, 53.2)	0.86 (0.85, 0.88) <0.001

Third or higher	24,711	83.3 (82.7, 83.9)	1.08 (1.07, 1.09) <0.001	20,368	53.8 (52.7, 54.9)	0.90 (0.88, 0.92) <0.001
Child age (in months)	87,854	§	1.00 (1.00, 1.00) <0.001	60,889	§	0.88 (0.87, 0.88) <0.001
Sex of child						
Male	45,596	80.4 (79.9, 80.9)	1.00	31,166	55.7 (54.8, 56.6)	1.00
Female	42,258	81.7 (81.2, 82.2)	1.01 (1.01, 1.02) <0.001	29,723	55.6 (54.7, 56.5)	1.01 (1.00, 1.03) 0.062
Household size	87,854	§	1.00 (1.00, 1.00) 0.049	60,889	§	1.00 (0.99, 1.00) 0.001
Social group						
Scheduled castes	70,067	80.2 (79.8, 80.6)	1.00	38,478	58.6 (57.8, 59.4)	1.00
Scheduled tribes	13,391	87.6 (86.7, 88.6)	1.06 (1.05, 1.07) <0.001	9,760	61.8 (59.8, 63.8)	1.08 (1.06, 1.10) <0.001
Others	4,396	86.1 (84.6, 87.6)	1.06 (1.04, 1.07) <0.001	12,651	44.5 (43.2, 45.8)	0.94 (0.91, 0.96) <0.001
Religion						
Hinduism	65,062	81.1 (80.6, 81.5)	1.00	44,601	56.8 (56.1, 57.5)	1.00
Islam	12,443	81.0 (80.0, 81.9)	0.99 (0.98, 1.00) 0.012	9,071	51.0 (49.4, 52.7)	0.95 (0.93, 0.97) <0.001
Others	10,349	80.6 (79.1, 82.1)	0.93 (0.92, 0.94) <0.001	7,217	52.0 (49.4, 54.7)	0.84 (0.81, 0.86) <0.001
Locality of residence						
Urban	17,404	79.1 (78.2, 79.9)	1.00	13,742	50.4 (48.9, 52.0)	1.00
Rural	70,450	81.7 (81.3, 82.1)	1.00 (0.99, 1.01) 0.408	47,147	57.3 (56.6, 58.0)	1.07 (1.05, 1.09) <0.001
Region of residence						
North	16,060	79.0 (78.2, 79.8)	1.00	12,316	54.7 (53.5, 56.0)	1.00
South	11,222	85.3 (84.5, 86.1)	1.07 (1.05, 1.08) <0.001	6,859	57.7 (55.9, 59.4)	1.08 (1.05, 1.10) <0.001
East	17,314	82.6 (81.9, 83.4)	1.01 (1.00, 1.02) 0.029	11,288	54.9 (53.5, 56.3)	1.00 (0.98, 1.02) 0.941
West	7,686	80.9 (79.4, 82.4)	1.02 (1.01, 1.04) <0.001	4,899	55.5 (53.0, 57.9)	1.03 (1.00, 1.06) 0.042
Central	23,077	77.2 (76.5, 77.9)	0.98 (0.97, 0.99) <0.001	16,949	55.3 (54.3, 56.3)	1.00 (0.98, 1.02) 0.787
Northeast	12,495	86.8 (85.9, 87.7)	1.02 (1.01, 1.04) 0.001	8,578	57.6 (55.6, 59.5)	1.01 (0.98, 1.04) 0.473
NFHS year‡						
1993	na	na	na	6,015	43.4 (41.6, 45.2)	1.00
1999	na	na	na	5,001	46.8 (44.8, 48.7)	1.06 (1.02, 1.11) 0.005
2006	na	na	na	4,630	46.3 (44.1, 48.4)	1.11 (1.07, 1.16) <0.001
2016	na	na	na	22,404	54.8 (53.7, 55.9)	1.33 (1.28, 1.39) <0.001
2021	87,854	81.0 (80.7, 81.4)	na	22,839	63.8 (62.8, 64.8)	1.47 (1.41, 1.53) <0.001

Note: Analysis based on pooled data from the five waves of the National Family Health Survey, 1993–2021.
† All samples are unweighted.
‡ Compared with Figure S1, the cumulative sample sizes across survey rounds may vary slightly because cases with missing values on key independent variables were excluded from the analysis.
§ Percentages were not calculated because this is a continuous (count) variable.
ANC: antenatal care, CI: confidence interval, na: not applicable, NFHS: National Family Health Survey, RR: relative risk

Table S7. Association of maternal education and household wealth with mixed feeding under six months and continued breastfeeding at 12-23 months in India.

	Mixed feeding under six months			Continued breastfeeding at 12-23 months		
	Sample (60,889)[†]	% (95% CI)	RR (95% CI) *p*	Sample (117,861)[†]	% (95% CI)	RR (95% CI) *p*
Maternal education						
No schooling	18,469	9.9 (9.3, 10.5)	1.00	36,194	84.9 (84.3, 85.4)	1.00
1st–5th grade	7,979	12.8 (11.6, 14.0)	1.11 (1.03, 1.20) 0.005	15,629	81.8 (80.9, 82.8)	0.98 (0.97, 0.99) <0.001
6th–8th grade	10,139	14.4 (13.1, 15.6)	1.12 (1.04, 1.20) 0.002	19,891	79.6 (78.7, 80.6)	0.98 (0.97, 0.99) <0.001
9th–12th grade	17,378	14.8 (13.9, 15.7)	1.15 (1.07, 1.23) <0.001	33,250	76.4 (75.6, 77.2)	0.98 (0.97, 0.99) <0.001
Above 12th grade	6,924	18.0 (16.5, 19.5)	1.30 (1.20, 1.42) <0.001	12,897	69.2 (67.9, 70.6)	0.94 (0.93, 0.96) <0.001
Household wealth						
Poorest	14,905	10.7 (9.9, 11.5)	1.00	28,148	87.1 (86.5, 87.7)	1.00
Poorer	13,764	11.5 (10.7, 12.4)	1.12 (1.04, 1.20) 0.002	25,733	84.4 (83.7, 85.1)	0.99 (0.98, 1.00) 0.004
Middle	12,137	13.3 (12.4, 14.3)	1.26 (1.17, 1.36) <0.001	23,649	79.7 (78.9, 80.5)	0.97 (0.97, 0.98) <0.001
Richer	11,023	15.0 (13.8, 16.2)	1.36 (1.25, 1.48) <0.001	21,863	74.0 (73.0, 75.0)	0.93 (0.92, 0.94) <0.001
Richest	9,060	18.4 (17.0, 19.8)	1.47 (1.34, 1.62) <0.001	18,468	67.5 (66.3, 68.7)	0.86 (0.85, 0.88) <0.001
Maternal age (year)						
15-19	5,680	10.3 (9.1, 11.6)	1.00	5,650	82.8 (81.3, 84.3)	1.00
20-24	24,832	12.6 (12.0, 13.3)	0.98 (0.89,1.06) 0.588	42,720	78.6 (78.0, 79.2)	0.97 (0.96, 0.99) <0.001
25-29	19,366	14.5 (13.7, 15.3)	1.03 (0.93,1.13) 0.595	42,022	78.7 (78.1, 79.4)	0.98 (0.97, 1.00) 0.034
30-34	7,660	15.4 (14.0, 16.8)	1.06 (0.96,1.18) 0.254	18,831	80.8 (79.8, 81.7)	1.00 (0.98, 1.01) 0.920
35-39	2,660	14.7 (12.1, 17.3)	1.11 (0.98,1.27) 0.106	6,704	82.8 (81.3, 84.4)	1.01 (0.99, 1.03) 0.297
40-44	559	14.0 (8.8, 19.3)	1.03 (0.81,1.31) 0.778	1,569	84.4 (81.3, 87.5)	1.00 (0.97, 1.02) 0.876
45-49	132	8.2 (0.7, 15.8)	1.13 (0.71,1.81) 0.597	365	84.2 (78.3, 90.2)	0.96 (0.91, 1.01) 0.086
Mass media exposure						
No	20,489	10.6 (10.0, 11.3)	1.00	38,643	84.3 (83.7, 84.8)	1.00
Yes	40,400	14.8 (14.2, 15.4)	1.02 (0.97, 1.08) 0.394	79,218	77.1 (76.6, 77.6)	1.02 (1.01, 1.02) <0.001
Number of ANC visits						
<4	32,092	12.1 (11.6, 12.7)	1.00	61,701	82.0 (81.5, 82.5)	1.00
≥4	28,797	14.7 (14.0, 15.4)	1.01 (0.96, 1.06) 0.689	56,160	76.8 (76.2, 77.4)	1.03 (1.02, 1.04) <0.001
Place of delivery						
Home/others	17,331	9.5 (8.9, 10.2)	1.00	33,549	84.9 (84.3, 85.4)	1.00
Healthcare facility	43,558	14.8 (14.3, 15.4)	1.18 (1.11, 1.26) <0.001	84,312	77.4 (77.0, 77.9)	0.99 (0.98, 1.00) 0.057
Birth order						
First	21,893	11.6 (10.9, 12.3)	1.00	41,302	75.6 (74.9, 76.3)	1.00
Second	18,628	16.5 (15.6, 17.4)	1.46 (1.38, 1.54) <0.001	37,046	79.3 (78.6, 80.0)	1.04 (1.03, 1.04) <0.001
Third or higher	20,368	12.3 (11.5, 13.0)	1.29 (1.21, 1.38) <0.001	39,513	84.0 (83.4, 84.5)	1.04 (1.03, 1.05) <0.001

Child age (in months)	60,889	§	1.20 (1.19, 1.22) <0.001	117,861	§	0.98 (0.98, 0.98) <0.001
Sex of child						
Male	31,166	13.8 (13.1, 14.4)	1.00	61,730	80.2 (79.7, 80.8)	1.00
Female	29,723	12.9 (12.3, 13.5)	0.94 (0.90, 0.98) 0.003	56,131	78.7 (78.1, 79.2)	0.98 (0.97 ,0.98) <0.001
Household size	60,889	§	1.01 (1.01, 1.02) <0.001	117,861	§	1.00 (1.00, 1.00) <0.001
Social group						
Scheduled castes	38,478	14.7 (14.1, 15.2)	1.00	74,638	78.9 (78.5, 79.4)	1.00
Scheduled tribes	9,760	7.7 (6.3, 9.2)	0.70 (0.64, 0.76) <0.001	18,284	84.4 (83.3, 85.4)	0.98 (0.98, 0.99) <0.001
Others	12,651	11.3 (10.4, 12.1)	1.05 (0.97,1.13) 0.202	24,939	79.5 (78.8, 80.3)	1.00 (0.99, 1.01) 0.764
Religion						
Hinduism	44,601	13.1 (12.6, 13.6)	1.00	86,019	80.0 (79.6, 80.4)	1.00
Islam	9,071	14.7 (13.5, 15.9)	1.08 (1.02, 1.14) 0.011	17,584	78.4 (77.4, 79.4)	0.96 (0.95, 0.97) <0.001
Others	7,217	13.3 (11.3, 15.2)	1.14 (1.05, 1.23) 0.001	14,258	75.0 (73.3, 76.7)	0.88 (0.87, 0.90) <0.001
Locality of residence						
Urban	13,742	17.2 (16.1, 18.4)	1.00	28,781	71.7 (70.8, 72.7)	1.00
Rural	47,147	12.1 (11.6, 12.5)	0.90 (0.86, 0.95) <0.001	89,080	82.3 (82.0, 82.7)	1.04 (1.03, 1.05) <0.001
Region of residence						
North	12,316	13.4 (12.5, 14.2)	1.00	22,668	77.4 (76.6, 78.2)	1.00
South	6,859	13.4 (12.2, 14.5)	0.91 (0.84, 0.98) 0.011	14,601	67.6 (66.5, 68.8)	0.89 (0.88, 0.90) <0.001
East	11,288	14.8 (13.8, 15.8)	1.16 (1.08, 1.24) <0.001	23,105	87.5 (86.9, 88.1)	1.08 (1.07, 1.09) <0.001
West	4,899	9.6 (8.0, 11.2)	0.76 (0.69, 0.83) <0.001	10,325	75.0 (73.6, 76.5)	0.96 (0.95, 0.97) <0.001
Central	16,949	13.9 (13.2, 14.6)	1.07 (1.01, 1.14) 0.025	30,062	81.7 (81.1, 82.2)	1.01 (1.00, 1.02) 0.016
Northeast	8,578	11.3 (10.1, 12.5)	1.08 (0.99, 1.17) 0.069	17,100	88.7 (87.9, 89.4)	1.08 (1.07, 1.09) <0.001
NFHS year‡						
1993	6,015	5.2 (4.4, 5.9)	1.00	11,523	79.2 (78.2, 80.2)	1.00
1999	5,001	4.5 (3.7, 5.4)	0.70 (0.60, 0.82) <0.001	9,371	80.8 (79.7, 81.9)	1.04 (1.03, 1.06) <0.001
2006	4,630	20.0 (18.3, 21.7)	3.16 (2.82, 3.53) <0.001	8,978	82.6 (81.4, 83.7)	1.05 (1.04, 1.07) <0.001
2016	22,404	15.4 (14.5, 16.2)	1.99 (1.75, 2.25) <0.001	46,056	79.9 (79.2, 80.5)	1.05 (1.04, 1.07) <0.001
2021	22,839	14.2 (13.4, 14.9)	1.88 (1.66, 2.14) <0.001	41,933	78.1 (77.5, 78.8)	1.03 (1.01, 1.04) <0.001

Note: Analysis based on pooled data from the five waves of the National Family Health Survey, 1993–2021.

† All samples are unweighted.

‡ Compared with Figure S1, the cumulative sample sizes across survey rounds may vary slightly because cases with missing values on key independent variables were excluded from the analysis.

§ Percentages were not calculated because this is a continuous (count) variable.

ANC: antenatal care, CI: confidence interval, NFHS: National Family Health Survey, RR: relative risk

Table S8. Prevalence of exclusive breastfeeding across states and union territories (UTs) in India as per the National Family Health Surveys (NFHSs) - 2019-2021 and 2023-2024.

State/UT	2019-2021[†]	2023-2024[‡]
Andaman and Nicobar Islands (UT)	75.8	85.6
Andhra Pradesh	69.0	69.5
Arunachal Pradesh	63.4	50.5
Assam	63.9	54.3
Bihar	59.0	62.5
Chandigarh (UT)	51.4	§
Chhattisgarh	80.4	75.8
Dadra & Nagar Haveli and Daman & Diu (UT)	80.0	64.1
Goa	61.4	§
Gujarat	65.4	71.4
Haryana	70.0	41.2
Himachal Pradesh	69.9	60.8
Jammu and Kashmir (UT)	62.0	59.2
Jharkhand	76.2	63.8
Karnataka	61.1	61.6
Kerala	55.2	72.7
Ladakh (UT)	70.9	52.1
Lakshadweep (UT)	66.0	60.8
Madhya Pradesh	74.3	56.4
Maharashtra	71.6	64.7
Manipur	70.8	¶
Meghalaya	43.1	46.8
Mizoram	67.9	64.9
Nagaland	43.7	37.4
NCT of Delhi (UT)	64.7	48.3
Odisha	72.9	66.7
Puducherry (UT)	64.7	73.1
Punjab	55.6	51.0
Rajasthan	70.6	54.3
Sikkim	28.3	49.6
Tamil Nadu	55.0	55.6
Telangana	69.5	64.8
Tripura	62.1	53.4
Uttar Pradesh	59.8	34.6
Uttarakhand	53.1	40.8
West Bengal	52.9	58.4
India	63.9	55.8

[†] Source: Rai, R. K., De Neve, J. W., Bromage, S., Kim, R., & Subramanian, S. V. (2026). Trends in breastfeeding indicators across Indian states and Union Territories (1993-2021): Estimating progress towards the 2030 Global Nutrition Target. *Preventive Medicine*, 209, 108600. https://doi.org/10.1016/j.ypmed.2026.108600.

[‡] Source: International Institute for Population Sciences (IIPS). (2026). National Family Health Survey (NFHS-6), 2023-24: India and State/UT Fact Sheets. Mumbai: IIPS.

[§] Percentages not shown in NFHS 2023–2024 fact sheet as the denominator has fewer than 25 unweighted cases.

[¶] Not reported in NFHS 2023–2024.

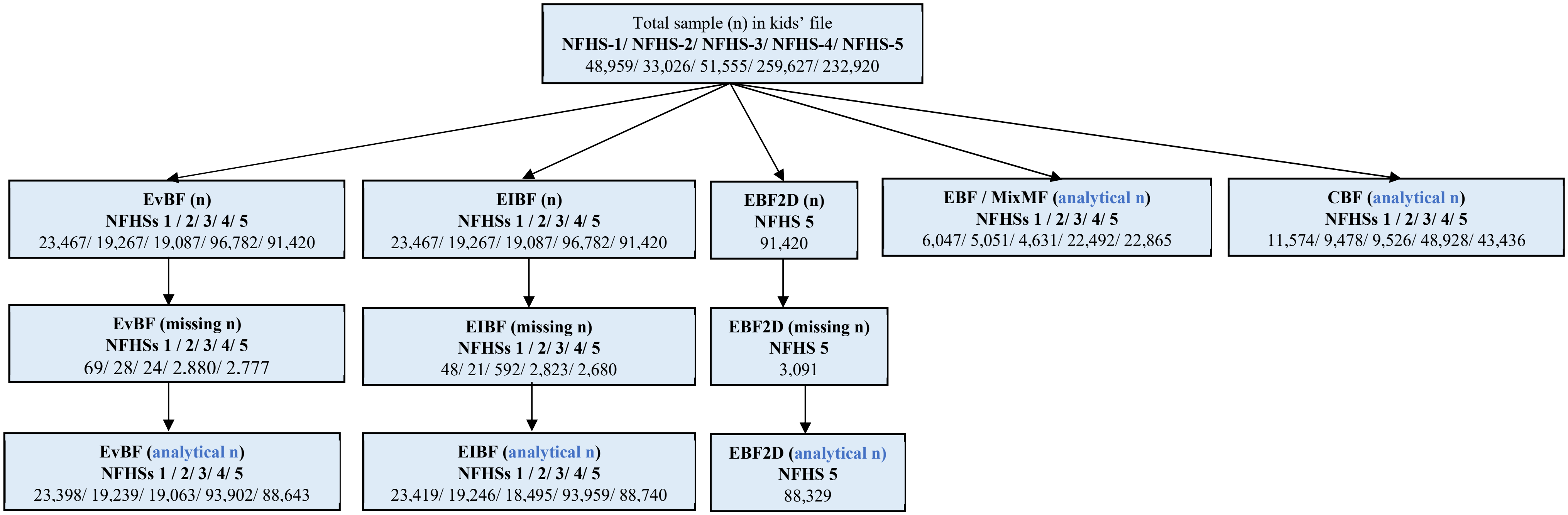

Figure S1. Schematic representation of the analytical sample (n) derivation process for breastfeeding indicators across five waves of the National Family Health Survey: NFHS-1 (1993), NFHS-2 (1999), NFHS-3 (2006), NFHS-4 (2016), and NFHS-5 (2021).

EvBF: ever breastfed, EIBF: early initiation of breastfeeding, EBF2D: exclusively breastfed for the first two days after birth, EBF: exclusive breastfeeding under six months, MixMF: mixed feeding under six months, CBF: continued breastfeeding 12-23 months, NFHS: National Family Health Survey

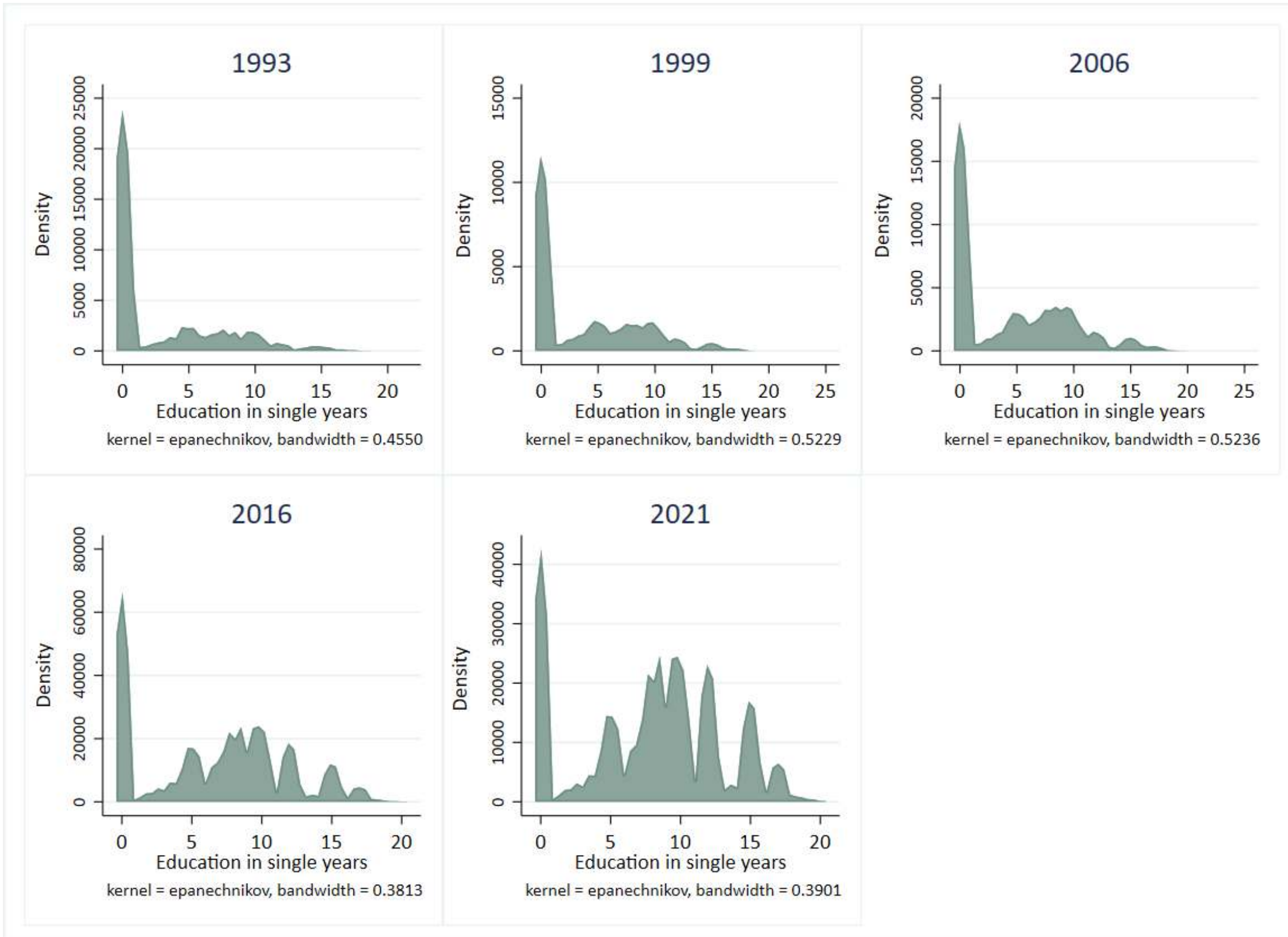

Figure S2. Kernel density estimates (weighted) of educational attainment (years of schooling) across five waves of the National Family Health Survey (NFHS), 1993–2021.

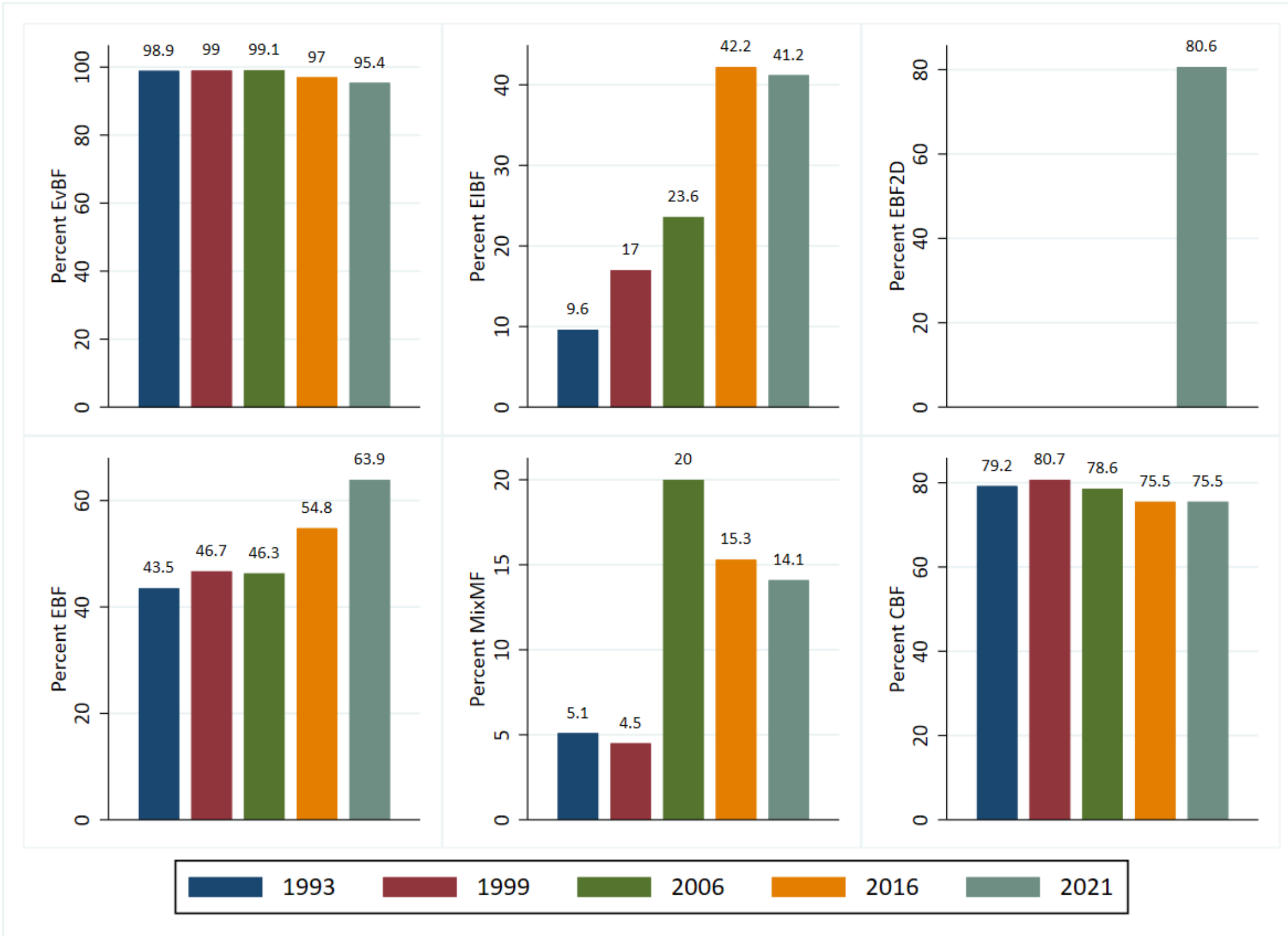

Figure S3. Trends in the prevalence of breastfeeding indicators: findings from the NFHS, 1993–2021.

EvBF: ever breastfed, EIBF: early initiation of breastfeeding, EBF2D: exclusively breastfed for the first two days after birth, EBF: exclusive breastfeeding under six months, MixMF: mixed feeding under six months, CBF: continued breastfeeding 12-23 months, NFHS: National Family Health Survey
